# Two monoclonal antibodies against glycoprotein Gn protect mice from Rift Valley Fever challenge by cooperative effects

**DOI:** 10.1371/journal.pntd.0008143

**Published:** 2020-03-11

**Authors:** Benjamin Gutjahr, Markus Keller, Melanie Rissmann, Felicitas von Arnim, Susanne Jäckel, Sven Reiche, Reiner Ulrich, Martin H. Groschup, Martin Eiden

**Affiliations:** 1 Institute of Novel and Emerging Infectious Diseases, Friedrich-Loeffler-Institut, Greifswald-Insel Riems, Germany; 2 Saxon State Laboratory of Health and Veterinary Affairs, Dresden, Germany; 3 Department of Experimental Animal Facilities and Biorisk Management, Friedrich-Loeffler-Institut, Greifswald-Insel Riems, Germany; 4 Institute of Veterinary Pathology, Leipzig University, Leipzig, Germany; School of Veterinary Medicine University of California Davis, UNITED STATES

## Abstract

Rift Valley fever virus (RVFV) is a zoonotic arbovirus that causes severe disease in humans and ruminants. The infection is characterized by abortions in pregnant animals, high mortality in neonates as well as febrile illness in humans that develop in 1% of cases encephalitis or hemorrhagic fever. There is presently no specific antiviral treatment for RVFV infection available. In this study, two monoclonal antibodies (mAbs), raised against glycoprotein Gn, were applied in a therapeutic study. Treatment of RVFV infected mice with neutralizing mAb Gn3 alone at two different time points (30 minutes before or 30 minutes after virus challenge) showed only moderate efficacy of about 58.3% survival in both applications. However, a combination therapy together with non-neutralizing mAb Gn32 demonstrated complete protection (100% survival) when applied 30 minutes after the lethal challenge dose. The increase of mAb efficacy is probably based on cooperative neutralization effects. These data suggest that a combination therapy with mAbs Gn3 and Gn32 could be an effective treatment option against RVFV infection.

## Author summary

Rift Valley fever virus represents an acute viral disease affecting animals especially livestock and humans and is responsible for widespread outbreaks throughout Africa and on the Arabian Peninsula. The virus causes abortions and high mortality especially in young animals, whereas the symptoms in humans range from mild flu-like illness to *severe* hemorrhagic manifestations that *can* be *lethal*. So far, no antiviral therapeutics for animals nor humans were available yet. Therefore, we evaluated two monoclonal antibodies—one neutralizing and one non-neutralizing—in a mouse model for therapeutic treatment against Rift Valley fever. We selected these antibodies since they exhibited cooperative effects *in vitro*. During Rift Valley fever virus infection in mice, the applied neutralizing antibody alone showed only partial protection. In contrast, a combined application with both antibodies, lead to a complete protection in one treatment group (100% survival). A detailed pathological and molecular analysis clearly indicated a strong reduction of virus replication in target tissues of treated mice. Taken together, these results identified two monoclonal antibodies with strong antiviral effects against Rift Valley fever infection, *which are promising candidates* for therapeutic interventions against RVFV.

## Introduction

Rift Valley fever virus (RVFV) belongs to the family *Phenuiviridae*, genus *Phlebovirus* and contains a single stranded tripartite genome of negative polarity. It consists of three segments that code for nucleocapsid protein (S-segment), two glycoproteins Gn, Gc (M-segment) and a RNA dependent RNA polymerase (L-segment). In addition, two non-structural proteins are encoded by the S-segment (NSs) and M-segment (NSm), respectively [1]. More than 50 mosquito species including *Anopheles*, *Aedes* and *Culex spp*. have been associated with RVFV transmission, either by isolation of the virus from field-collected mosquitoes or by successful experimental transmission in laboratory setting [2, 3].During inter-epizootic periods, the virus may persist by vertical transmission in *Aedes* mosquitoes and the deposition of infected eggs. Subsequent heavy rainfalls and flooding lead to hatching of RVFV infected vectors causing epidemics. The virus emerged during the 1970ths and 1980ths throughout Africa and entered the Arabian Peninsula in 2000 [4]. Main focus with re-occurrent epidemics are Sub-Saharan countries (e.g. Mauritania), South-Africa, Tanzania, Kenya and Egypt [4]. Based on this emergence and due to numerous susceptible mosquito species that are also found in Europe, there is a significant risk for introduction and subsequent dissemination of RVFV in Europe [5].

Especially in pregnant ruminants (sheep, goats and cattle), the virus causes high abortion rates up to 100% in sheep and displays a similar mortality in infected newborn lambs. Generally increasing age reduces susceptibility, but adult animals can also be affected and suffer from weakness, anorexia, diarrhea, bloody nasal discharge and jaundice [6]. Apart from abortions, no clinical symptoms can be observed in camels; however, (hyper) acute clinical courses are described in an outbreak in Northern Mauritania [7]. Human infection mainly results from direct contact or inhalation during the handling of infected animals and tissues. Most infections display non-specific flu-like symptoms [8]. Severe cases are associated mostly with ocular complications (e.g. retinitis, reduced vision) and in 1–2% of the cases with jaundice and hemorrhagic manifestations. The course is normally preceded by a febrile phase of 3 to 4 days including temporal recovery or long-lasting fever up to 10 days [9]. Severe complications can be also associated with encephalitis [10–12] Up to now no approved antiviral therapeutics nor approved vaccine for Rift Valley fever is available [13]. Known antivirals like Ribavirin or Favipiravir showed only limited efficacy in animal models [14]. Additional small molecules are currently still under development [14]. Most recently, rabbit and human derived monoclonal antibodies against glycoprotein Gn were shown to be protective in mice against Rift Valley fever infections [15, 16]. Gn is a type I transmembrane protein which forms together with glycoprotein Gc non-covalently linked heterodimers on the lipid bilayer envelope of the virion and allows virus attachment, uptake into cells and Gc mediated cell fusion. The Gn protein consists of an N-terminal ectodomain and a C-terminal transmembrane domain followed by a cytoplasmic tail. The ectodomain is main target for neutralizing antibodies and composed of three domains including an N-terminal helical domain, followed by a β-ribbon and a small globular domain [17].

In our study, we analyzed the therapeutic application of two murine mAbs raised against glycoprotein Gn in a mouse model for Rift Valley fever. The combined application of a neutralizing with a non-neutralizing antibody led to complete protection against an otherwise lethal dose of RVFV attributed to cooperative interference. These results open up new therapeutic treatment possibilities.

## Materials and methods

### Protein expression and purification

Both antibodies were generated by immunization of BALB/c mice with bacterial expressed glycoprotein Gn which encompasses 429 amino acids of the ectodomain (residues 154–582, UniProt accession no. P21401) fused to a N-terminal His-tag. For this purpose the RVFV MP-12 derived sequence was cloned into vector pET19b followed by expression in *E*.*coli* BL21 cells. Resulting inclusion bodies were lysed under denaturating conditions including 8M urea and purified over Nickel NTA chelating agarose columns (Qiagen, Hilden, Germany). Refolding of the protein was done by dialysis in 50 mM sodium carbonate/bicarbonate buffer (pH = 9.6). The resulting protein fractions were analyzed via SDS-PAGE [18].

### Antibody generation and purification

BALB/c mice were immunized intraperitoneally with 50 μg of recombinant purified Gn protein suspended in complete Freund’s adjuvant and repeated three times at intervals of about 4 weeks. This was followed by an immunization without adjuvant. Finally, mouse spleen cells were fused with SP2/0 myeloma cells and resulting hybridoma cells were assessed for mAb against RVFV glycoprotein Gn using a Gn-based indirect IgG ELISA. Both mAb were reactive in ELISA, western blot and indirect immunofluorescence [19]. Purification of mAb was carried out with HiTrap Protein G antibody purification columns according to manufacturer’s instructions (GE Healthcare Bio-Sciences, Uppsala, Sweden).

### Virus and cell culture

Rift Valley fever strain 35/74 (accession number: JF784386-88) was propagated in BHK-21 cells (Collection of Cell Lines in Veterinary Medicine, Friedrich-Loeffler-Institut, Germany). RVFV strain 35/74 was isolated from a liver of a sheep that died during an RVFV outbreak in the Free State province of South Africa in 1974 [20] The virus titer was determined using a 50% Tissue Culture Infective Dose (TCID_50_) assay, calculated as described by Spearman and Kärber [21]. Briefly, 100 μl of 10-fold serial diluted strain 35/74 were added to 90% confluent monolayers of BHK-21 cells. After incubation at 37°C, 5% CO2 for 6 days, plates were fixed with neutral buffered formalin and stained with crystal violet.

Virus titration: Samples tested positive in quantitative real-time RT-PCR (RT-qPCR) were used for virus isolation on BHK-21 cells. Corresponding homogenates were diluted from 10^−1^–10^−8^ in Minimum Essential Medium (MEM) supplemented with penicillin-streptomycin and 2% fetal calf serum (FCS). 100μl of each dilution was added in quadruplicates on a 96 well cell plate seeded with a 90% confluent monolayer of BHK-21 cells. After incubation for 1h at 37°C, 5% CO2, each well was overlaid with 100μl medium, incubated at 37°C, 5% CO2 for 6 days and subsequently fixed with neutral buffered formalin and stained with crystal violet.

### Animals and experimental design

For the mouse challenge model, 3–6 month old BALB/c mice were transferred from in-house specific-pathogen-free (SPF) facility into BSL-3 containment and kept in isolated ventilated cages. The experiments were approved by the competent authority of the Federal State of Mecklenburg-Western Pomerania, Germany, on the basis of national and European legislation, namely EURL 63/2010 for the protection of animals used for experiments (LALLF 7221.3–1.1-048/17). Following an initial clinical examination, all animals have been accustomed to the new surroundings prior to the initiation of experiments. All animals were fed with commercial feed and had access to water ad libitum.

In total 102 mice were included in this study and allocated in 6 groups with equal proportion of male and female individuals. 96 mice were inoculated intraperitoneally with 200μl 10^2.7^ TCID_50_/ml of RVFV strain 35/74. Six control animals were treated only with mAbs (mAb control group). From 96 infected mice, 24 individuals were treated with PBS (PBS group) and 72 individuals with different antibody regimen. Antibodies (200μg in 100μl PBS) were applied intravenously in the tail vein either 30 minutes before virus challenge (timepoint T1) or 30 min after virus challenge timepoint T2). Accordingly mice were treated at time point T1 with mAb Gn3 (n = 12, Gn3 _T1 group), at time point T2 (n = 12, Gn3_T2 group) as well as at time point T1 with mAb Gn32 (n = 12, Gn32 _T1 group) and at time point T2 (n = 12, Gn32_T2 group). In addition a simultaneous administration of mAb Gn3 and Gn32—again short before challenge (n = 12, Gn3+Gn32_T1 group) or after virus application (n = 12, Gn3+Gn32_T2 group), referred as “combi groups”.

Blood samples were taken daily by submandibular venipuncture from corresponding mice starting from 1 dpi till 13 dpi. Mice behavior and clinical anomalies were scored twice daily by a defined score sheet (S1 Fig). The animals were inspected for the presence of signs of disease including ruffled fur, lethargy, immobility and bleedings. Depending on the course of the disease, the listed symptoms can occur in individual degrees of severity. Corresponding values were added in an individual score sheet. The humane endpoint was defined as a cumulative score ≥8. Mice were euthanized with an overdose of Isofluran CP 1ml/ml (CP-Pharma, Burgdorf, Germany) followed by heart puncture at 13 dpi or when termination criteria from the score sheet were met earlier.

### Molecular detection of RVFV-specific RNA

RNA was extracted from liver, brain and blood cruor with NucleoMag VET Kit (MACHEREY-NAGEL, Düren, Germany) and King Fisher Flex Purification System (Thermo Scientific, Waltham, USA), according to the manufacturer´s instructions. As internal extraction as well as PCR control, MS2 bacteriophage was added to each sample to exclude false negative results [22]. The presence of RVFV-derived RNA was determined by a quantitative real-time RT-PCR (RT-qPCR) [23]. Used primer (RVF-forv:5'-TGAAAATTCCTGAGACACATGG-3'; RVF-rev: 5'-ACTTCCT TGCATCATCTGATG-3') and probe (RVF-probe: 5'-FAM-CAATGTAAGGGGCC TGTGTGGACTTGTG-BHQ1-3’) target the L-segment of RVFV at nucleotide position 2912–3001. MS2 bacteriophage derived RNA was detected via primers MS2F (5’-CTCTGAGAGCGGCTCTATTGGT-3’), MS2R (5’-GTTCCCTACAACG AGCCTAAATTC-3’) and MS2probe (5’-HEX-TCAGACACGCGGTCCGCTATAACGA-BHQ1-3’). A synthetic RNA comprising the target region of the RT-qPCR was utilized as calibrator for quantification. The synthetic calibrator was generated by in-vitro transcription from corresponding DNA-sequence, which contains at 5’-end an additional T7 promotor sequence for in vitro transcription. [24].

### Serology

Monoclonal IgG1 antibodies Gn3 and Gn32 were raised against recombinant, bacterially (*E*.*coli*) expressed ectodomain of RVFV glycoprotein Gn and were reactive in ELISA, indirect immunofluorescence (IIFA) and Western blot [19]. Neutralizing activity of mAb Gn3 and mAb Gn3 in combination with Gn32 was determined in a serum neutralization assay (SNT) using RVF vaccine strain MP-12 [25]. Briefly, 100 TCID_50_ of MP-12 was added to duplicates of serial twofold diluted mAbs. Following an incubation of 30 min at 37°C and 5% CO2, 3 x 10^5^ Vero 76 cells (Collection of Cell Lines in Veterinary Medicine, Friedrich-Loeffler-Institut, Germany) were added to each well. Plates were incubated at 37°C, 5% CO2 for 6 days. Neutralizing doses of 50% (ND_50_) were expressed as the reciprocal of the serum dilution that still inhibited > 50% of cytopathogenic effect. Furthermore, dilution series of mAbs and heat inactivated sera of heart puncture was tested in the same SNT protocol but using strain 35/74 on BHK-21 cells. As a positive control neutralizing serum of a cattle immunized with recombinant, bacterially (*E*.*coli*) expressed ectodomain of RVFV glycoprotein Gn and as negative control serum of a naive sheep were used.

Mouse sera were analyzed with the ID Screen RVFV competition multi-species ELISA (ID Vet, Montpellier, France) according to the manufacturer’s instructions. The ELISA is based on the detection of antibodies (IgM/IgG) against the RVFV nucleoprotein NP.

### Epitope mapping

A peptide library of RVFV Gn was generated by synthesizing oligopeptides with 20 amino acid length and 12 amino acid overlap by JPT (Berlin, Germany). Peptide assay was similar performed as already described in Lagatie et al. [26]. In short, NUNC Immobilizer Streptavidin plates (Thermo Scientific, Waltham, USA) were coated with 2.5μM/well peptides at 4°C overnight. After that, plates were blocked with 3% skimmed milk for 1h, room temperature. Furthermore, plates were incubated with monoclonal antibodies (1:100 diluted in blocking buffer) for 1h, 37°C. After that, secondary goat anti mouse HRP antibody (Dianova, Hamburg, Germany) was used as detection of specific binding mAbs and incubated for 1h, 37°C. Finally, TMB (3.3′,5.5′-Tetramethylbenzidin) was added and incubated for 10min. Reaction was stopped with 1M sulphuric acid and read out in an ELISA reader at 450nm. Threefold washing steps with tris- buffered saline and 0.1% Tween 20 was done between single steps.

### Pathological examination and immunohistochemistry

Necropsy was performed according to standard procedures under a work bench at biosafety level 3 (BSL-3) conditions. Specimen from brain, lung, liver and spleen were fixed during necropsy in 4% neutral buffered formaldehyde for at least more than 21 days, processed, embedded in paraffin wax, sectioned at 2–4 μm thickness, and stained with hematoxylin and eosin. The grade of characteristic histopathological lesions (hepatitis, encephalitis, lymphoid depletion, follicular hyperplasia) was semi-quantitatively assessed on a 0–3 scale as follows: 0 = no obvious findings, 1 = mild, 2 = moderate and 3 = severe findings.

Immunohistology was performed with the avidin–biotin–peroxidase complex method (ABC, Elite PK6100; Vector Laboratories, Burlingame, CA, USA) with 3-amino-9-ethylcarbazole (AEC, Dako, Glostrup, Denmark) as chromogen and hematoxylin counterstain. The primary antibody used for the detection of RVFV nucleoprotein was a heat-inactivated serum of a sheep immunized with recombinant RVFV MP12-strain nucleoprotein (internal code: S24NP) in a dilution of 1:4000. As positive control, Vero 76 cells were infected with MP-12 strain, pelleted by centrifugation (1500 rpm, 20 min, 12°C) 24 h post infection, and processed and embedded in paraffin wax similar to the tissue samples. Negative controls consisted of uninfected Vero 76 cells and replacement of the primary antibody by sera of a non-immunized sheep on serial sections, respectively. The distribution of the RVFV antigen was semi-quantitatively assessed for each organ on a 0–3 scale as follows: 0 = no viral antigen, 1 = focal or oligofocal, 2 = multifocal, and 3 = confluent to diffuse immunoreactive cells. Pathological examination and immunohistochemistry of samples from the Gn32 (T1/T2) group was not performed.

### Native immunofluorescence

For native IFA an in-house protocol was used as follows: Complete medium from MP-12 infected Vero 76 cells—grown on cover slips- was removed and cells were washed three times with ice cold phosphate buffered saline (PBS) including 1mM CaCl_2_ and 0.5mM MgCl_2_ (PBS++). Subsequently cells were incubated with mAb Gn3 or Gn32 diluted 1:5 in 0.35% bovine serum albumin (BSA) in PBS++ at 4°C for 60 minutes. After washing three times with ice cold PBS++ the secondary antibody (Cy3-labeld goat anti mouse, Jackson Immunoresearch, USA), diluted with 0.35% (BSA) in PBS++, was added (diluted 1:2500) for 45 minutes at 4°C. After washing three times with ice cold PBS++, cells were fixed with ice cold methanol/acetone (1:1) for 10 minutes at 4°C. Following the third washing step (three times with ice cold PBS ++), staining with 4′,6-diamidino-2-phenylindole (Carl Roth, Germany)—diluted 1:20000 in PBS–was performed for 10 minutes at 37°C. The final washing step was carried out twice with ice cold PBS++ and a final washing step with pure water. Each cover slip was mounted on slides using elvanol (Thermo Scientific, Waltham, USA). Fluorescence staining was evaluated by fluorescence microscopy (Nikon, Japan).

### Data and statistical analysis

EC_50_ and IC_50_ values were obtained by fitting the data to a 4-Parameter Logistic Regression model [27] using the nplr package in R. Frederic Commo and Brian M. Bot (2016). nplr: N-Parameter Logistic Regression. R package version 0.1–7 (R Core Team (2018). R: A language and environment for statistical computing. R Foundation for Statistical computing, Vienna, Austria). CI and DRI values were obtained by CompuSyn software (ComboSyn, Inc., Paramus, NJ, USA) to quantify synergy.

Kaplan-Meier survival curves were analyzed by the log rank test and virological data (viral RNA, virus titration and pathological scores) compared using an ANOVA (Kruskal-Wallis H) test and correlation was done with Pearson product moment correlation with SigmaPlot (Systat Software, San Jose, CA, USA). Data were graphed using Microsoft Excel 2016 (Microsoft corporation, Redmond, Wa, USA). See also S1 Data.

## Results

### In vitro characterization of mAbs Gn3 and Gn32

**Fig 1:**
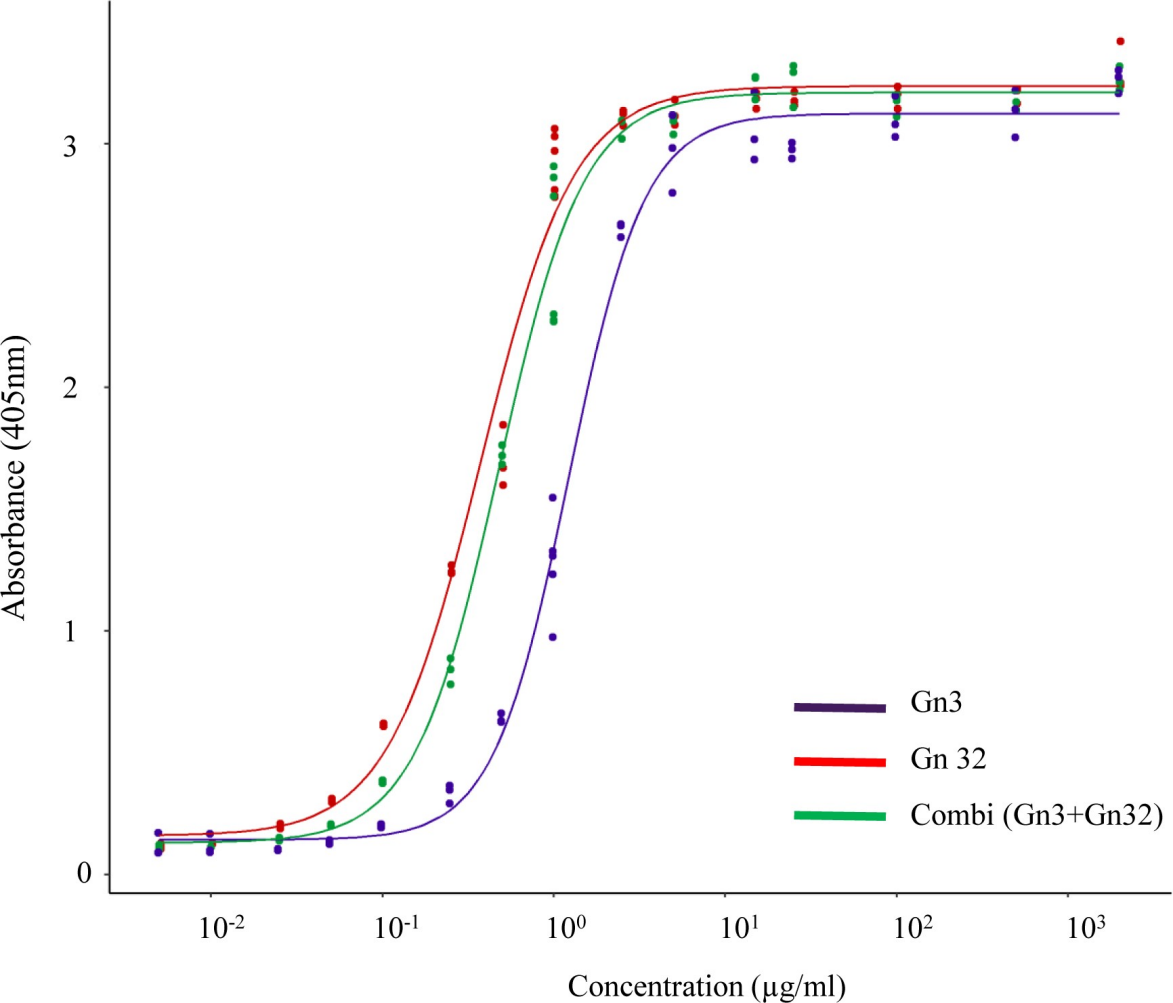
ELISA analysis of mAbs Gn3 and Gn32 using recombinant glycoprotein Gn as antigen. Experiments were repeated three times.

**Fig 2:**
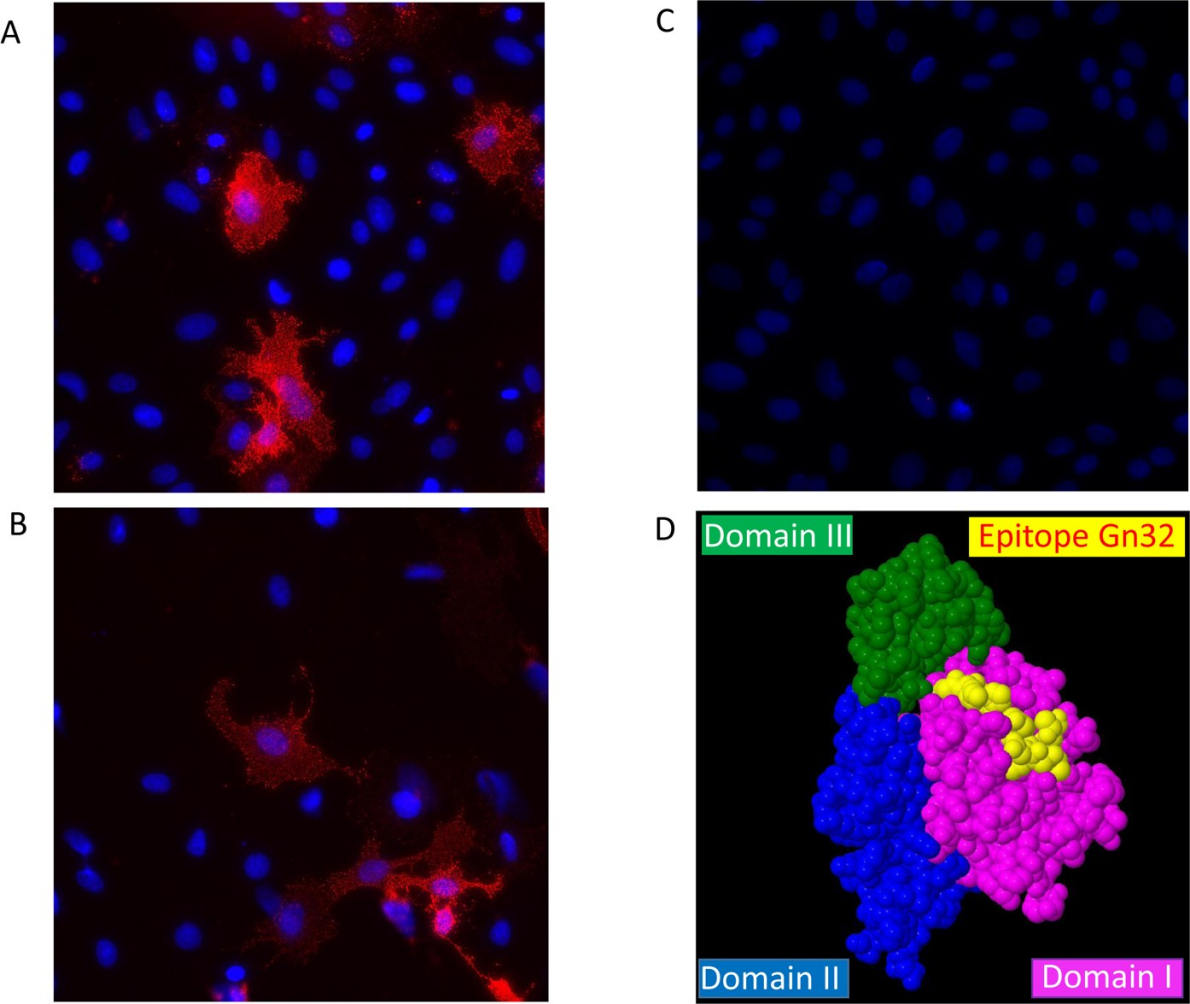
Detection of mAb using native indirect immunofluorescence assay and a 3D-model of Gn32 derived epitope. Binding of glycoprotein Gn by monoclonal antibodies Gn3 (A) and Gn32 (B) on surface of MP-12 infected cells was visualized with Cy3-labeled secondary antibody. Mock infected cells (C). Cell nuclei were stained with DAPI (magnification 40x). **(D)** Epitope of Gn32 (displayed in yellow) is located on the surface of glycoprotein Gn (PDB 5Y0Y) in a spacefill 3D-model (Geneious Prime, Auckland, New Zealand, Version 2019.2.3).

The binding of both monoclonal antibodies Gn3 and Gn32 to recombinant RVFV Gn ectodomain was assessed in ELISA yielding EC_50_ values of about 1.29 μg/ml and 0.38 μg/ml (Fig 1). Co-incubation of both mAbs showed a shift to an increased binding activity (EC_50_ = 0.49 μg/ml) probably based on cooperative interactions. An epitope mapping study on mAbs Gn3 and Gn32 was performed using biotinylated oligopeptides with 20 amino acid (aa) length and 12 aa overlap covering the whole sequence of glycoprotein Gn ectodomain. Gn32 targeted two adjacent peptides at the outermost n-terminus of Gn protein (encompassing sequence: PGKGHNYIDGMT, S2 Data; Fig 2D) in contrast to Gn3 that did not react with any of the linear epitopes.

The binding of both mAb to glycoprotein Gn was further assessed by native indirect immunofluorescence demonstrating physiological recognition of corresponding Gn3 and Gn32 epitopes evolving from MP-12 infected Vero cells (Fig 2A and 2B).

**Fig 3:**
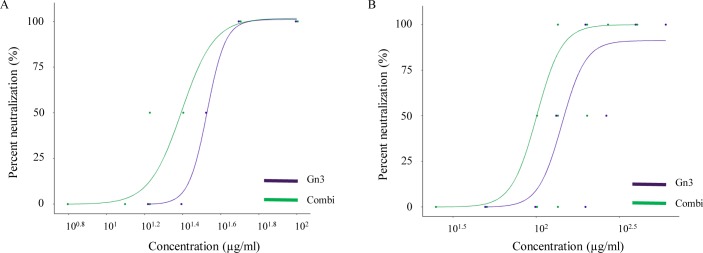
mAb Gn3 and Gn32 neutralize RVFV in two serum neutralization tests. Data are shown as percentage neutralization and a regression model is fitted using 4-Parameter Logistic. **(A)** SNT using RVFV- strain MP-12. **(B)** SNT using RVFV- strain 35/74 Experiments were performed in duplicate.

Both mAbs were also assessed in serum neutralization test (Fig 3). Only mAb Gn3 exhibited neutralizing activity and reached a 50% neutralizing concentration (IC_50_) against RVFV vaccine strain MP-12 at 33.0 μg/ml. As already seen in ELISA, co-incubation of mAbs Gn3 with Gn32 showed a synergistic effect and reduced IC_50_ to 24.6 μg/ml (Fig 3A). IC_50_ values of mAb Gn3 against virulent RVFV strain 35/74 were 4–5 times higher showing a concentration of 147.2 μg/ml and in combination with Gn32 of 100.6 μg/ml (Fig 3B, S3 Data). The synergistic effects of both mAbs were additionally quantified by so-called combination index (CI) and dose-reduction index (DRI) as described earlier [28]. The calculation was carried out by CompuSyn software which basically quantifies synergetic effects of drug combinations [29, 30]. The CI, which is divided into 3 categories (<1: synergistic effects, 1: additive effects, >1: antagonistic effects) revealed F(a)_55_ (Fraction affected) values of 0.39 and 0.31 for MP-12 and strain 35/74 respectively. The DRI indicates how many folds of dose-reduction for each compound at a given effect are allowed in synergistic combination. The corresponding values were 2.59 for MP-12 and 3.17 for strain 35/74 (detailed data used for calculation and CI and DRI at other F(a) are listed in S4 Data).

### In vivo protective efficacy of mAbs Gn3 and Gn32 against lethal RVFV challenge

**Fig 4:**
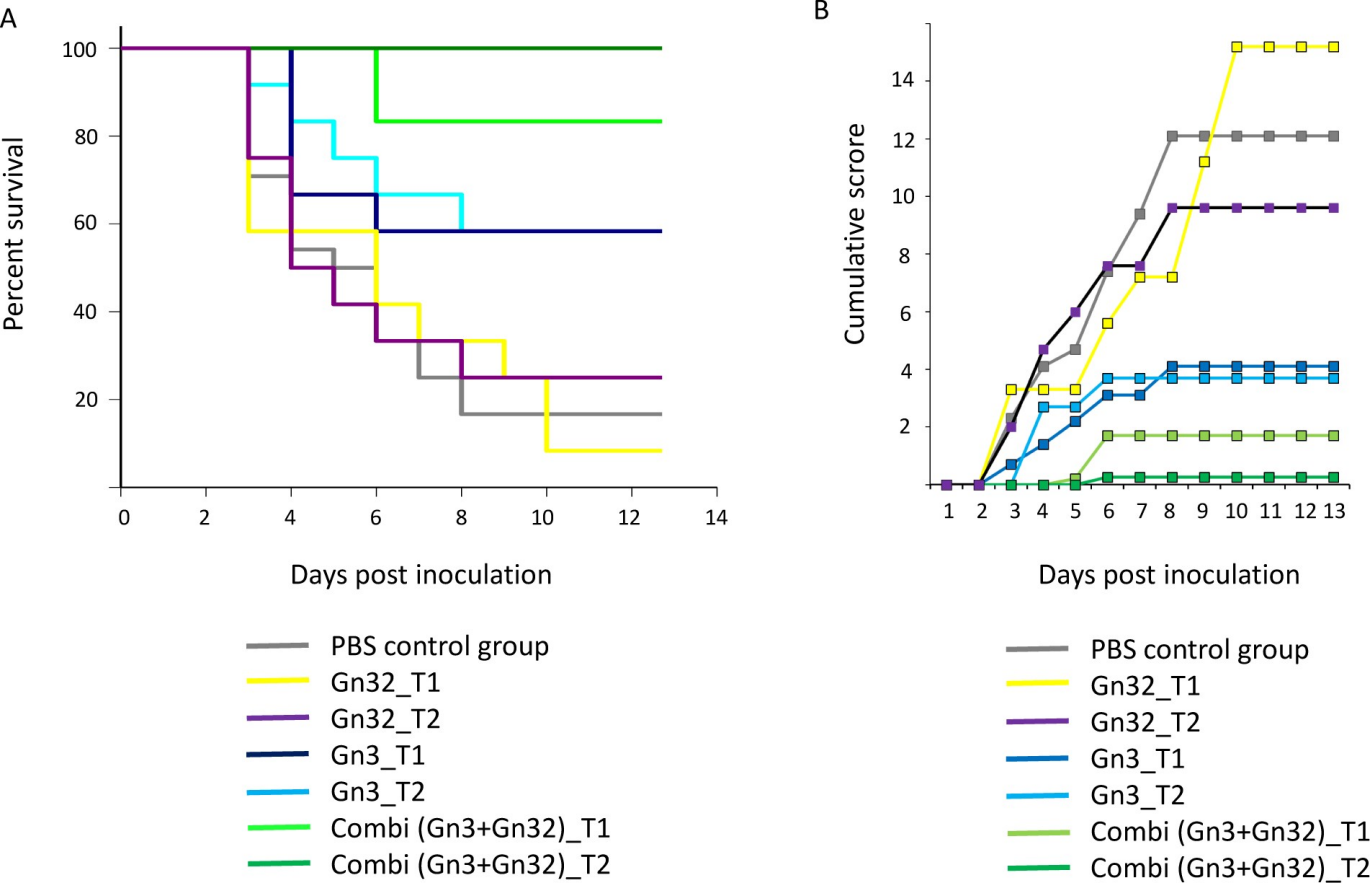
Survival curves and cumulative mean clinical scores of tested groups. **(A)** Efficacy of antibody protection was assessed by survival analysis. (A) Kaplan-Meier log rank test was performed; n.s. between mAb-Gn3-treated (T1/T2) and PBS control, p<0.001 between mAb-Gn3-Gn32-treated (T1) and PBS control, p<0.001 between mAb-Gn3-Gn32-treated (T2) and PBS control, n.s. between mAb-Gn3-Gn32-treated (T1/T2) and Gn3 (T1/T2). **(B)** Clinical scores were monitored and recorded daily after infection. n.s. not significant.

To determine whether mAbs Gn3 and Gn32 had therapeutic activity, BALB/c mice inoculated i.p. with RVFV strain 35/74, were subjected to different antibody treatments using applications 30 minutes before (T1 groups) or 30 minutes after (T2 groups) virus challenge studies were carried out each with Gn3 and Gn32 alone or in combination with both (combi groups). Unfortunately, one mouse of T2 combi group died during antibody application. Treatment with Gn32 alone (n = 12 in both groups) exhibited no effects (8% and 25% survival in T1 and T2 group respectively) and treatment with Gn3 in RVF infected mice (n = 12 in both T1/T2 groups) showed medium efficacy (about 58.3% survival in both applications). However, a combination therapy together with non-neutralizing mAb Gn32 demonstrated nearly complete protection (83.3% survival, T1 group) and a subsequent trial (application 30 min after virus challenge, T2 group) protected 100% of the animals from a lethal challenge dose (Fig 4A). In this group, only one individual displayed a transient clinical sign (ruffled fur). In contrast, the PBS control group presented a survival rate of only 16.67%. Diseased mice showed typical signs like ruffled fur, lethargy and acute death around 3–6 days post inoculation (dpi) and signs of neurological infection on 8 dpi, when there was no effective antibody protection. Mean clinical score of corresponding groups is depicted in Fig 4B. The compilation of individual scores is found in S5 Data. Non-infected mice (mAb control group) showed no signs of antibody incompatibility (S5 Data).

**Fig 5:**
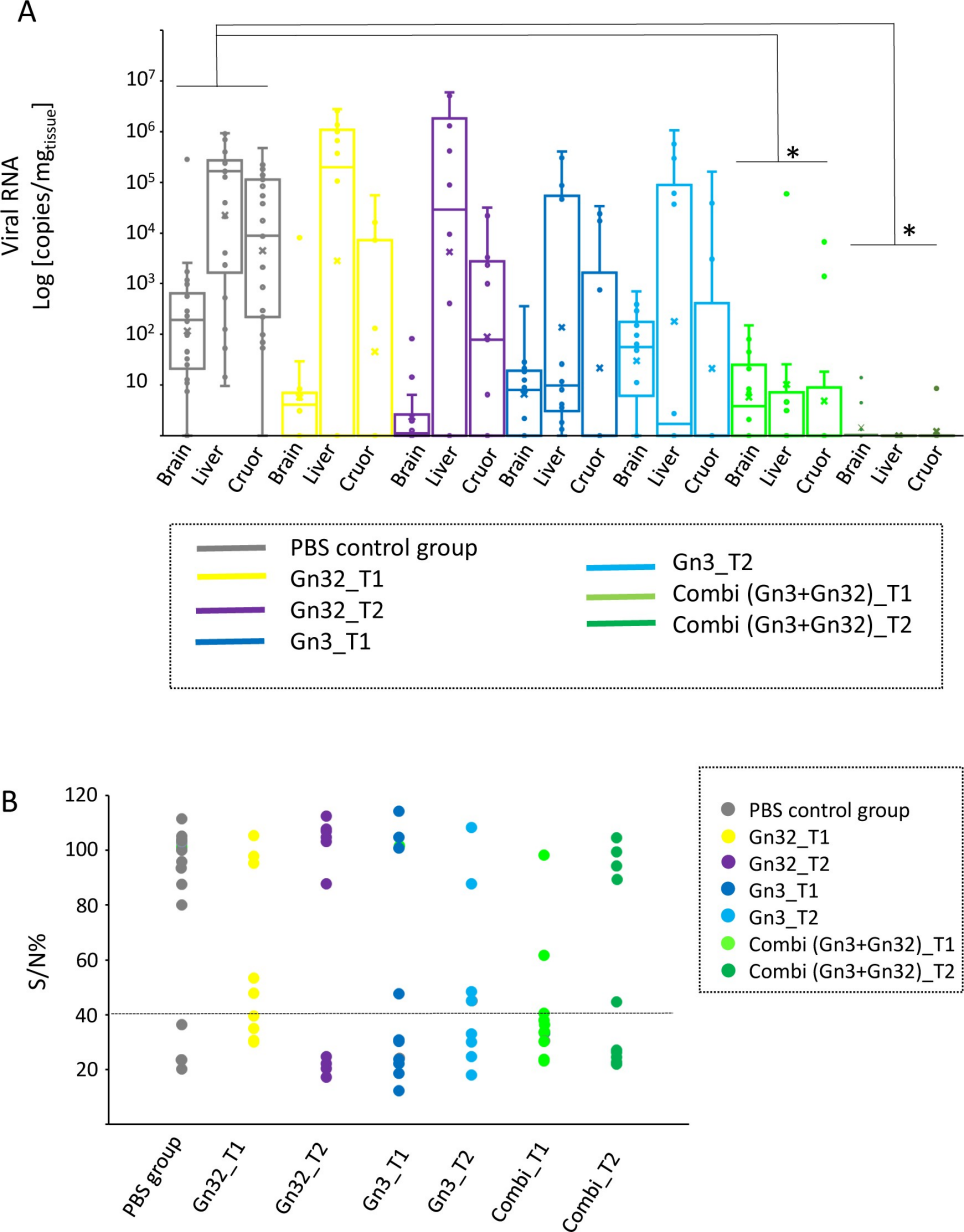
PCR results of brain, liver and cruor and ELISA results of serum from heart blood of individual mice. **(A)** Virus loads were assessed by quantitative real-time RT-PCR with RVFV specific primers and quantified with a synthetic calibrator in samples from liver, brain and cruor. The results represent the virus loads [log10 copies (mg tissue)] of each group in a box blot diagram. Significance was analyzed by ANOVA (Kruskal-Wallis H) test (*p<0.05) **(B)** Antibodies in mice serum samples at day of death were determined by a commercial competition ELISA kit targeting antibodies against nucleocapsid protein. Results are shown as sample-to-positive ratio for each individual mouse. Horizontal line marks ELISA cut off.

Analysis of tissues (brain and liver) as well as cruor in necropsied mice showed a significant reduction of viral RNA in combined mAb treated animals, compared to the untreated group (Fig 5A). Main target tissue was liver with an average value of 2.3x10^5^ copies/mg tissue, followed by cruor (7.6x10^4^ copies/mg tissue) and brain (1.2x10^4^ copies/mg tissue) in the untreated group. A strong decline was observed in both Gn3 groups as well as the combi T1 group regarding cruor samples as well as in Gn32 -T1/T2, Gn3 T1 and combi T1 groups regarding brain. However, high viral RNA loads remained in the liver tissues of the Gn32 (mean values 7.4x10^5^-1.6x10^6^ copies/mg tissue) and Gn3 groups (mean values: 7x10^4^-1.7x10^5^ copies/mg tissue). In contrast, no viral RNA was detected in liver of the combi T2 group and low amounts of RNA (mean value: 1 copy/mg) in the brain of 3 individuals as well as 1.8 copies/mg tissue in cruor of one animal indicating a nearly complete protection.

In addition, all PCR positive samples were used for virus titration on BHK-21 cells (S2 Fig). The results demonstrate a high correlation with the PCR results. In particular, there was no live virus in tissues from the combi T1 group. A compilation for all individuals is seen in S6 Data.

The daily blood samples revealed viremia in the PBS group during 2–6 dpi in PCR and virus titration. All Gn3 and combined mAb treated groups showed a shortened viremic phase, whereas both Gn3 groups still having peaks similar to those of the PBS group. The combi groups showed delayed viremia and especially combi T2 group showed a distinct lower viral load during viremic phase. Gn32 groups showed a longer viremia, whereas peaks and duration are lower than the PBS group.See detailed data in S7 Data.

Sera from necropsied animals were further tested in a competition ELISA (Fig 5B). The lowest number of seropositive samples (16.7%) were observed in the PBS group probably due to rapid virus spread and onset on disease. Interestingly in both T1 groups (Gn3 and combi group) a distinct higher number of animals were seropositive (58.3% and 75%, respectively) compared to the T2 group (33% and 54.5%, respectively). No such differences were seen in the Gn32 groups (44% vs 40%).

Finally, sera were tested for neutralizing activity, which correlated in most cases with ELISA positive results. The titer ranged from 1:10 up to 1:60 (see S6 Data summarizing complete individual serological and molecular data).

**Fig 6:**
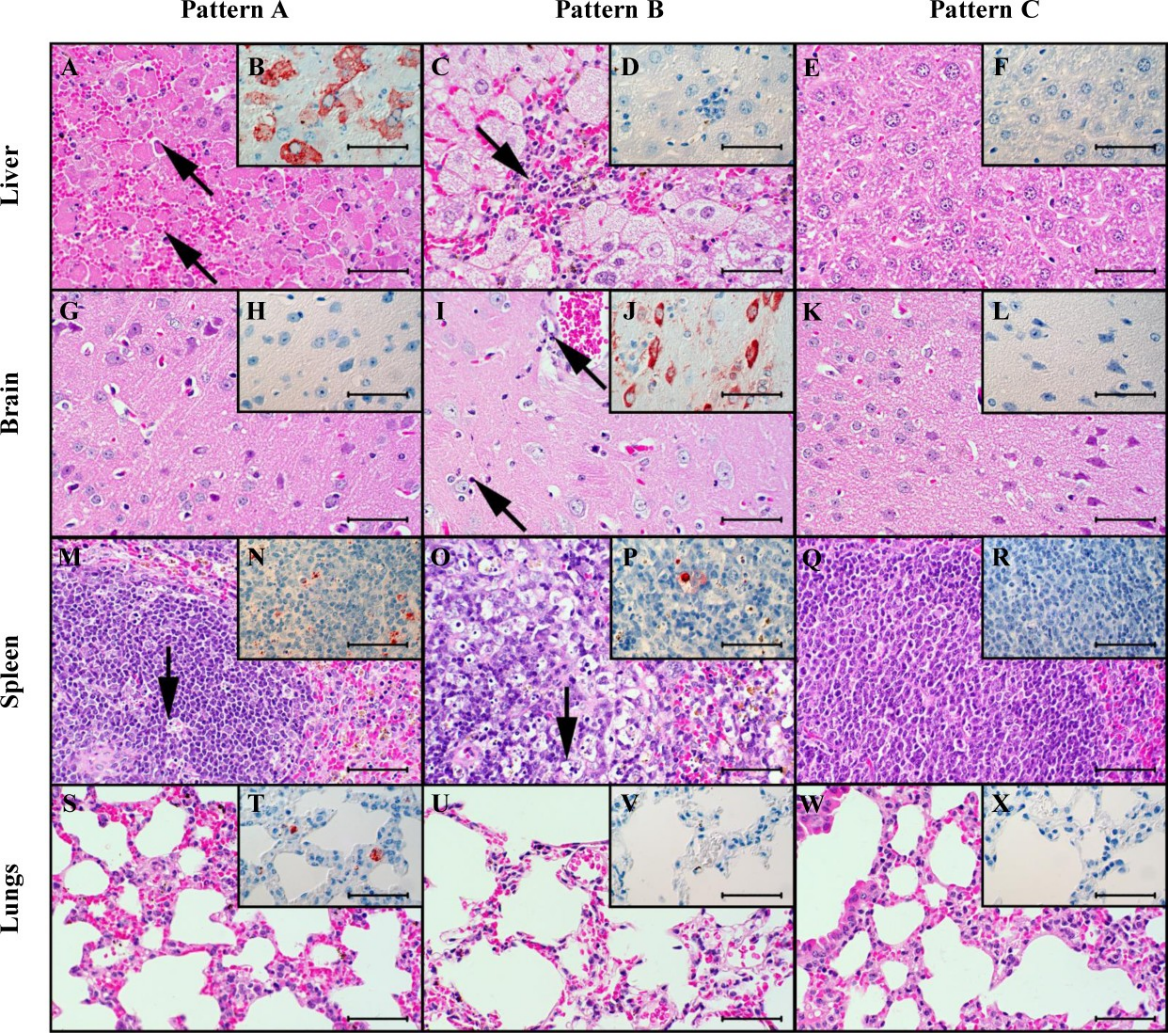
Histopathological and immunohistochemical findings in different tissues of three individual mice. Light microscopy revealed three principally different patterns of lesions and antigen distribution in the livers (first row, A-F), brains (second row, G-L), spleens (third row, M-R), and lungs (fourth row, S-X) of mice which succumbed due to the infection within the first 6 days (left column: P18-865, mouse #25, 3 dpi, A, B, G, H, M, N, S, T) later in the time course of the disease (middle column: P18-870, mouse #30, 8 dpi, C, D, I, J, O, P, U, V) and those which survived until the end of the observation period (right column, P18-878, mouse #78, 13 dpi, E, F, K, L, W, X and P18-851, mouse #68, 13 dpi Q,R). A.) The mice dying until 6 dpi display mainly a severe, acute, diffuse, necrotizing hepatitis with Councilman corpuscles (arrows), suggestive of hepatocellular apoptosis. B.) These mice show coalescing to diffuse antigen within hepatocytes. M.) There are few apoptotic or necrotic lymphocytes in the white matter of the spleen (arrow). C.) The few mice dying later than 6 dpi display multifocal infiltrations of macrophages and lymphocytes (arrow) and hepatocellular vacuolar degeneration within the livers. D.) There is no antigen present within the liver. I.) There are oligofocal necrotic neurons and glia (arrows) within the brain, interpreted as necrotizing polioencephalitis. J.) There is multifocal, neuronal antigen accumulation. O.) There are many apoptotic or necrotic lymphocytes (arrow) leading to lymphatic depletion within the spleen. Q.) There is follicular hyperplasia with enlarged follicles with a pale lymphoblast-rich center and a darker peripheral zone of more differentiated lymphocytes. A, C, E, G, I, K, M, O, Q, S, U, W.) hematoxylin-eosin. B, D, F, H, J, L, N, P, R, T, V, X.) Immunohistochemistry employing the avidin-biotin-peroxidase-complex method for RVFV nucleoprotein with AEC as chromogen (red-brown) and hematoxylin counterstain (blue). A-X.) Bars = 50 μm.

Necropsied animals were subjected to comprehensive histopathological and immunohistochemical analysis including semiquantitative assessment of virus antigen (nucleoprotein) distribution in brain, liver, spleen and lung (S3 Fig). Furthermore, the severity of the typical lesions was assessed employing four different histopathological scores (hepatitis, encephalitis, lymphoid depletion and follicular hyperplasia within the spleen). Thereby, three basic combinations of pathological findings and antigen distribution were observed (Fig 6): Pattern A (acute, high-grade hepatitis) includes animals with acute RVF infection that show predominantly high-grade, acute, confluent to diffuse, necrotizing hepatitis with Councilman corpuscles and intranuclear, irregular, eosinophilic inclusion bodies (Cowdry type B). Furthermore, these mice showed low to moderate lymphoid necrosis, apoptosis and/or depletion of the white pulp of the spleen. Pattern A is mostly found in individuals of the untreated PBS group, which are characterized by accumulation of RVF antigen mainly in liver and spleen. Main target cells for viral antigen were hepatocytes and macrophages in the red pulp and follicular dendritic cells in the spleen. In the lung, alveolar macrophages or interstitial and intravascular phagocytes were stained whereas in brain primarily neurons and rarely microglia were antigen positive.

Pattern B was observed in only few mice, which survived the acute, diffuse, necrotizing hepatitis and succumbed around 8 dpi. They exhibited a more subacute hepatitis with more pronounced infiltrates of macrophages and heterophils, and an acute, focal to multifocal, necrotizing polioencephalitis. Notably there was no RVFV antigen in the livers of these mice at the time of necropsy but in the lesioned areas within the brain. These mice also display lymphatic apoptosis, necrosis and depletion in the spleen.

Pattern C was observed in surviving animals and is characterized mainly by no obvious findings or low-grade, subacute, multifocal, periportal and randomized, lymphohistiocytic infiltrates with infrequent heterophils in the liver. Furthermore, some of these mice displayed low- to moderate follicular hyperplasia of the white pulp in the spleen. These findings were observed in the combi groups, especially the T1 group. Mice of the T2 group showed a significant lower incidence of hepatitis or were asymptomatic. Both groups exhibited a considerably lower antigen signal in liver, a lower incidence of lymphoid depletion but a significant higher incidence of follicular hyperplasia compared to the PBS group. The findings are summarized in Table 1.

**Table 1:** The frequency distribution of the lesion patterns in the different experimental groups shows a trend to a more favourable outcome (pattern C) in the combined treatment groups.

Group	Pattern		
	A	B	C
Gn3 T1 group	33.33%	8.33%	58.33%
Gn3 T2 group	41.67%	16.67%	41.67%
Gn3+Gn32 combi T1 group	16.67%	16.67%	66.67%
Gn3+Gn32 combi T2 group	0%	9.09%	90.91%
PBS group	70.83%	8.33%	20.83%

Pattern A: “Early-onset fatal hepatitis” characterized by severe, acute, coalescing to diffuse hepatitis and RVFV-antigen mainly in hepatocytes, as well as lymphocytic depletion; Pattern B: “Late-onset fatal polioencephalitis” characterized by mild to moderate, acute, focal to multifocal, necrotizing polioencephalitis and RVFV-antigen within neuroglial cells, as well as variable, subacute hepatitis and lymphocytic depletion; Pattern C: “Survivor with reactive lymphoid hyperplasia” characterized by mild to moderate, subacute to chronic, follicular hyperplasia in the spleen, as well as mild, subacute hepatitis or periportal infiltration and/or no obvious lesions.

Mice treated only with single mAb Gn3 showed in both (T1 and T2) groups mixed histopathological phenotypes or no findings at all. In general, a reduction of viral antigen was observed especially in the liver. The Gn3 T2 group showed also a significant lower incidence of lymphoid depletion compared to the PBS treated mice. Individual data regarding IHC and histopathology are deposited in S6 Data.

## Discussion

In this study, RVF neutralizing mAb Gn3 in combination with Gn32 was applied in a mouse RVF infection model demonstrating complete protection against virus challenge. It is the first trial about *in vivo* protection of cooperative mouse monoclonal antibodies against RVF. Similar antibody prevention studies against RVF were carried out very recently by applying human [16] or rabbit [15] derived mAbs. Furthermore, we conducted a detailed in-depth molecular and histo-pathological analysis of antibody treatment in infected mice.

MAbs Gn3 and Gn32 (as well as mixtures of both) showed high binding affinity to Gn in ELISA which are in the same EC_50_ range as RVFV-neutralizing rabbit mAbs [15]. In contrast, neutralizing activity exhibited IC_50_ values of about 100μg/ml that are significantly lower compared to rabbit antibodies (~3.0μg/ml) and human antibodies which were effective in the nanogram range [16]. However, the values are only comparable to a limited extent, since different neutralization or alternative assays were used (SNT vs plaque reduction neutralization test vs FACS based assays). In both reported cases, single mAb application led to protection against RVFV infection similar to the combined mAb treatment outlined here. Interestingly, for an efficient neutralization of virulent challenge strain 35/74 a distinctly higher antibody concentration was needed compared to vaccine strain MP-12, although both strains exhibited only 4 amino acid differences within glycoprotein Gn sequence (S4 Fig). These different amino acids are not located near the Gn32 derived epitope but three out of 4 amino acids induce slight modifications within the secondary structure of the strains, which could be responsible for the observed differences.

Via Pepscan analysis the main epitope of non-neutralizing mAb Gn32 could be successfully elucidated. The corresponding sequence PGKGHNYIDGMT is located in domain I of Gn and directly neighbors antigenic site A described in Wang et al. (16). Gn3 showed no distinct signal in Pepscan, indicating a conformational epitope. Even though Gn32 has no neutralizing activity *in vitro*, it increases significantly the neutralizing potency of Gn3. This synergistic effect was supported by the CompuSyn software with values of a CI of 0.39/ 0.31 and a DRI of 2.59/ 3.17. Similar effects were already described from studies with mAbs targeting other viruses, e.g. HIV triple and quadriple mAb combinations with CI of 0.2–0.8 and DRI of 2–39.9 [31]. Synergistic effects of RVFV neutralizing antibodies were previously reported from *in vitro* studies [32].

We therefor hypothesize that the mechanistic basis for this “cooperative neutralization” [28] is based on the binding of Gn32 to glycoprotein Gn which induces a conformational change and makes the epitope of mAb Gn3 more accessible. Thus, the induced conformational change allows a comprehensive and more effective binding of mAb Gn3. A similar cooperative effect of non-neutralizing mAbs that improve the activity of otherwise poorly neutralizing mAbs has been originally described for mAbs against HIV [31]. In any case, domain I of RVFV Gn plays an important role in antibody-mediated neutralization and protection, since additional neutralizing mAbs against RVFV cluster in this domain [16]. Application of Gn3 alone protected more than half of the group against a severe RVFV infection. In combination with non-neutralizing Gn32, 83% of the T1 group survived, whereas treatment 30 min after infection (T2) provided complete protection.

Complete protection was correlated with a significant reduction of viral load in all tested tissues and the incapability of further replication. The combined T2 antibody regimen could even completely eliminate the virus from the liver, which represents the main target organ of RVFV. Especially in the unprotected PBS group, strain 35/74 causes an infection with marked liver tropism, leading to a fulminant acute diffuse necrotizing hepatitis that has already been shown with other RVFV strains in mice [33, 34]. Importantly, all antibody treated groups showed a decrease and shortening in viremia. PBS group showed a viremia from 2–6 dpi, as reported earlier [33]. Especially in combi groups, viremia was clearly reduced, shortened and further delayed.

Our findings are principally consistent with two previous publications, where complete protection was reported by monoclonal antibodies but with significant differences regarding sources and mode of action [15, 16]. In the first case rabbit monoclonal antibodies were generated with high neutralizing activity targeting an epitope at domain B of Gn glycoprotein (corresponding to Gn domain II), which probably interferes with Gn/Gc heterodimer formation and subsequent cell fusion. In contrast, Wu et al. [16] isolated monoclonal antibodies from a recovered human patient that targeted epitopes at domain I of Gn glycoprotein and inhibited attachment of the virion to the cells.

Therefor further detailed studies are needed to elucidate the molecular Gn3/Gn32 mechanism of neutralization e.g. by measuring the binding kinetics of the antibody-antigen interaction and determining the crystal structure of corresponding mAb in complex with glycoprotein Gn.

Detailed histopathological and immunohistological analyses elucidated specific differences during the course of RVFV infection. In acute cases, coalescing to diffuse RVFV antigen was detectable mainly in hepatocytes, but also in multifocal macrophages and follicular dendritic cells in the spleen. The livers displayed characteristic severe, acute, coalescing to diffuse necrotizing hepatitis, and many hepatocytes displayed morphological changes reminiscent of apoptotic cell death as well as intranuclear Cowdry type B inclusion bodies [35]. These typical findings in the liver and spleen have been already noted during studies with other RVFV strains [34]. Furthermore, a moderate lymphoid depletion in the spleen was seen, which may lead to lymphopenia, and is probably part of a fatal systemic inflammatory response syndrome as a consequence of fulminant hepatitis. Lymphocyte apoptosis in spleens of infected mice that had died 3 dpi was reported previously [33]. These findings, which mainly occurred in the PBS group, were confirmed by a strong positive correlation between lymphoid depletion and the incidence of hepatitis (Pearson product moment correlation, r = 0.922; p = 0.0258* (*significant p<0.05)), RVFV-positive liver (r = 0.815; p = 0.0927) and cruor (r = 0.877; p = 0.0509).

MAb application indeed revealed a clearly differentiated picture of disease progression: Mice that survived the RVFV infection showed mainly only low-grade, multifocal, lymphohistiocytic lesions in the liver, together with follicular hyperplasia in the spleen. MAbs could not completely neutralize the virus, but minimized viral replication, which could be successfully repelled by a successful and strong immune response in the mouse. This led to lymphocytosis as found in vaccine [36] and mouse infection studies [33] in form of follicular hyperplasia. Therefore antibody treatment is characterized by a strong positive correlation between follicular hyperplasia and positive ELISA (r = 0.975; p = 0.00484*) and SNT (r = 0.921; p = 0.0261*) values and by a negative correlation with hepatitis (r = - 0.888; p = 0.0445*), virus replication in liver (r = - 0.925; p = 0.0242*) and viremia (r = - 0.93; p = 0.0219*). These findings were mainly seen in the combi groups, where a significantly low-grade hepatitis and frequent follicular hyperplasia was observed.

In summary, this study demonstrated an efficient protection against RVFV by application of a combined mAb regimen in a RVF mouse challenge model. Further studies will now try to determine the specific epitope of Gn3 antibody to find a comprehensive molecular explanation for the cooperative and protective effects. In addition, both murine mAbs will be converted into humanized or fully human variants to finally evaluate them in other species and to assess well-known side effects and treatment failures [37].

## Supporting information

S1 Fig Clinical score sheet for daily examination of infected mice. (TIF)

S2 Fig Virus replication from PCR positive tissues, determined as virus titer (log[TCID_50_/ml]). (TIF)

S3 Fig **Liver, spleen, brain and lung were examined for virus in IHC (A) and histopathology (B).** The organs of each mouse were evaluated according to a score between 0 and 3. Scores of each group were presented in a box plot diagram. Significance was analyzed by ANOVA (Kruskal-Wallis H) test (*p<0.05).
(TIF)

S4 Fig Alignment of amino acid sequences of RVFV strain 35/74 and MP-12. Red boxes indicates amino acid differences. Green box epitope of mAb Gn32. Protein secondary structure (e.g. alpha helix, turns and beta strand) was predicted by EMBOSS 6.5.7 using the original Garnier Osguthorpe Robson algorithm (GOR I) provided by the EMBOSS suite (http://emboss.sourceforge.net/).
(TIF)

S1 Data Statistical overview. (PDF)

S2 Data Epitope mapping of mAbs Gn3 and Gn32. (PDF)

S3 Data EC_50_ and IC_50_ values, obtained by fitting the data to a 4-Parameter Logistic Regression model using the nplr package in R. (PDF)

S4 Data CI and DRI data. (PDF)

S5 Data Clinical score of individual mice. (PDF)

S6 Data Individual PCR and virus titration values of different tissues (brain, liver) and cruor as well as individual serological data and pathological scores of examined tissues in IHC and histopathology (brain, liver, spleen and lung). (PDF)

S7 Data Figure and individual PCR and virus titration values of daily blood samples. (PDF)
