# Supplementary figures and images for: Two monoclonal antibodies against glycoprotein Gn protect mice from Rift Valley Fever challenge by cooperative effects
Source: PLoS Negl Trop Dis. 2020 Mar 11;14(3):e0008143. doi: 10.1371/journal.pntd.0008143 (PMC7089562; doi:10.1371/journal.pntd.0008143)

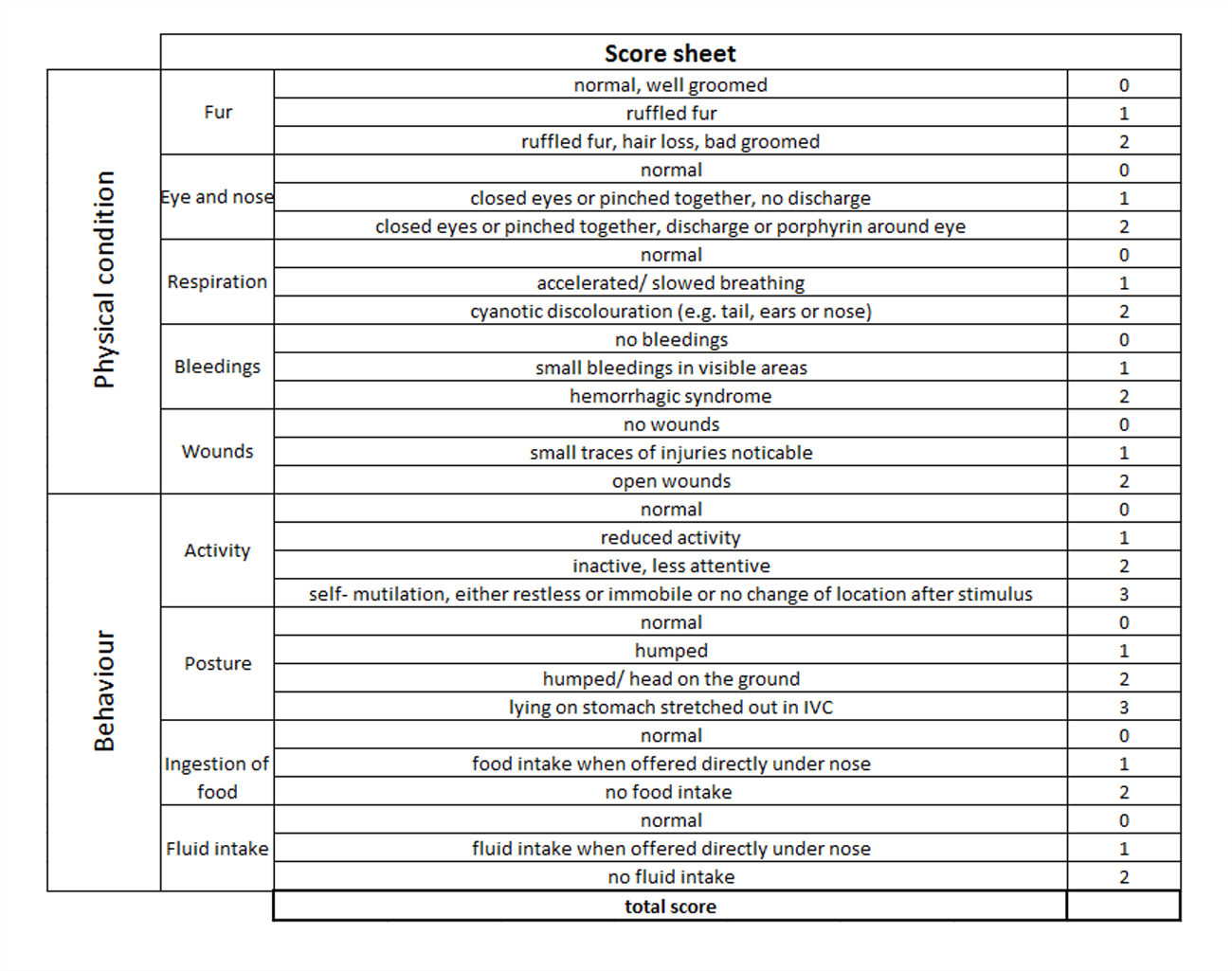

Supplement: S1 Fig — (TIF) [file pntd.0008143.s001.tif]

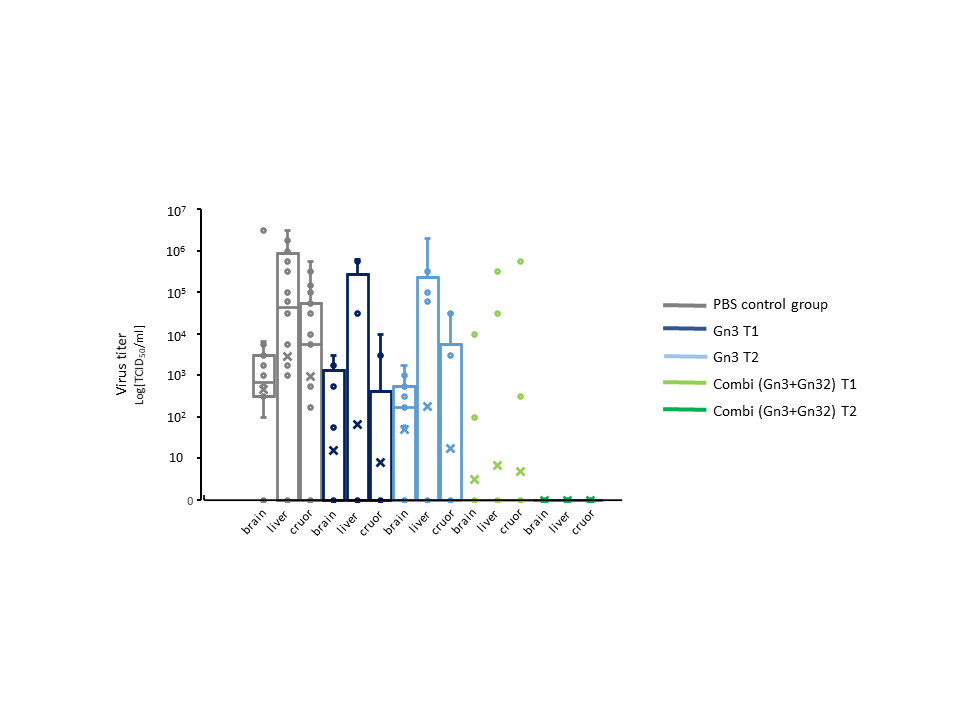

Supplement: S2 Fig — (TIF) [file pntd.0008143.s002.tif]

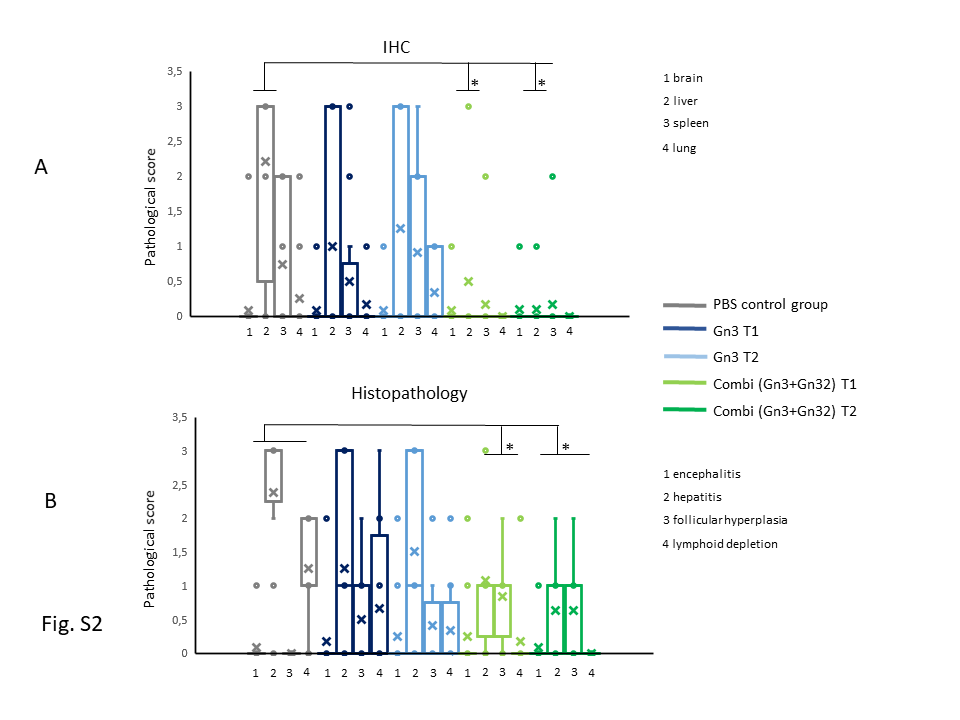

Supplement: S3 Fig — Liver, spleen, brain and lung were examined for virus in IHC (A) and histopathology (B). The organs of each mouse were evaluated according to a score between 0 and 3. Scores of each group were presented in a box plot diagram. Significance was analyzed by ANOVA (Kruskal-Wallis H) test (*p<0.05). (TIF) [file pntd.0008143.s003.tif]

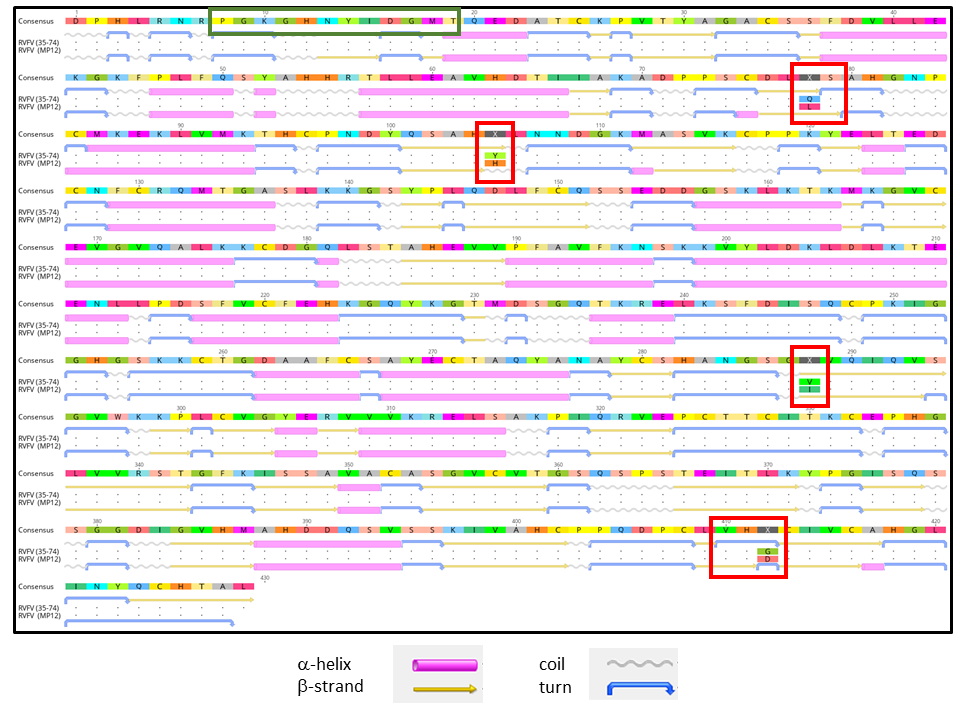

Supplement: S4 Fig — Red boxes indicates amino acid differences. Green box epitope of mAb Gn32. Protein secondary structure (e.g. alpha helix, turns and beta strand) was predicted by EMBOSS 6.5.7 using the original Garnier Osguthorpe Robson algorithm (GOR I) provided by the EMBOSS suite (http://emboss.sourceforge.net/). (TIF) [file pntd.0008143.s004.tif]
