# Supplementary material for: Two monoclonal antibodies against glycoprotein Gn protect mice from Rift Valley Fever challenge by cooperative effects
Source: PLoS Negl Trop Dis. 2020 Mar 11;14(3):e0008143. doi: 10.1371/journal.pntd.0008143 (PMC7089562; doi:10.1371/journal.pntd.0008143)
Supplement: S1 Data — (PDF) [file pntd.0008143.s005.pdf]

The log rank statistic for the survival curves (P = <0,001).

Survival curve (Fig. 3A)

column 1	PBS group
column 2	Gn3 T1
column 3	Gn3 T2
column 4	Gn3+Gn32 combi T1 group
column 5	Gn3+Gn32 combi T2 group

All Pairwise Multiple Comparison Procedures (Holm-Sidak method):

Overall significance level = 0,05

Comparisons	Statistic	P Value	Significant?
Column 1 vs. Column 5	18,064	0,000214	Yes
Column 1 vs. Column 4	13,681	0,00195	Yes
Column 1 vs. Column 2	5,77	0,123	No
Column 3 vs. Column 5	5,616	0,118	No
Column 3 vs. Column 2	5,581	0,104	No
Column 1 vs. Column 3	5,232	0,106	No
Column 3 vs. Column 4	2,14	0,462	No
Column 2 vs. Column 4	2,038	0,393	No
Column 4 vs. Column 5	1,921	0,304	No
Column 2 vs. Column 3	0,000682	0,979	No

Kruskal-Wallis One Way Analysis of Variance on Ranks

All Pairwise Multiple Comparison Procedures (Dunn's Method) :

Viral RNA (Fig. 5A)

column 1	PBS group
column 2	Gn3 T1
column 3	Gn3 T2

column 4	Gn3+Gn32 combi T1 group
column 5	Gn3+Gn32 combi T2 group

brain

Comparison	Diff of Ranks	Q	P<0,05
Col 1 vs Col 5	33,784	4,495	Yes
Col 1 vs Col 4	21,708	2,975	Yes
Col 1 vs Col 2	20,625	2,826	Yes
Col 1 vs Col 3	8,792	1,205	No
Col 3 vs Col 5	24,992	2,901	Yes
Col 3 vs Col 4	12,917	1,533	No
Col 3 vs Col 2	11,833	1,404	Do Not Test
Col 2 vs Col 5	13,159	1,527	No
Col 2 vs Col 4	1,083	0,129	Do Not Test
Col 4 vs Col 5	12,076	1,402	Do Not Test

liver

Comparison	Diff of Ranks	Q	P<0,05
Col 1 vs Col 5	40,083	5,233	Yes
Col 1 vs Col 4	28,792	4,002	Yes
Col 1 vs Col 3	18,375	2,554	No
Col 1 vs Col 2	13,25	1,842	Do Not Test
Col 2 vs Col 5	26,833	3,079	Yes
Col 2 vs Col 4	15,542	1,871	No
Col 2 vs Col 3	5,125	0,617	Do Not Test
Col 3 vs Col 5	21,708	2,491	No
Col 3 vs Col 4	10,417	1,254	Do Not Test
Col 4 vs Col 5	11,292	1,296	Do Not Test

cruor

Comparison	Diff of Ranks	Q	P<0,05
Col 1 vs Col 5	31,225	4,37	Yes

Col 1 vs Col 4	26,202	3,772	Yes
Col 1 vs Col 2	23,202	3,34	Yes
Col 1 vs Col 3	21,552	2,922	Yes
Col 3 vs Col 5	9,673	1,153	No
Col 3 vs Col 4	4,65	0,566	Do Not Test
Col 3 vs Col 2	1,65	0,201	Do Not Test
Col 2 vs Col 5	8,023	1,001	Do Not Test
Col 2 vs Col 4	3	0,383	Do Not Test
Col 4 vs Col 5	5,023	0,627	Do Not Test

Kruskal-Wallis One Way Analysis of Variance on Ranks
All Pairwise Multiple Comparison Procedures (Dunn's Method) :

Pathological scoring (Fig. S5A and B)

liver

Comparison	Diff of Ranks	Q	P<0,05		
Col 19 vs Col 31	24,862	3,308	Yes	Col 7,10,11,12	Combi T1
Col 7 vs Col 31	20,688	2,835	Yes	Col 19,22,23,24	Combi T2
Col 55 vs Col 31	14,604	2,001	No	Col 31.34,35,36	PBS
Col 43 vs Col 31	11,563	1,584	Do Not Test	Col 43, 46,47,48	Gn3 T1
				Col 55,58,59,60	Gn3 T2

lymphoid depletion

Comparison	Diff of Ranks	Q	P<0,05
Col 22 vs Col 34	27,109	3,634	Yes
Col 10 vs Col 34	23,817	3,286	Yes
Col 46 vs Col 34	19,317	2,665	Yes
Col 58 vs Col 34	14,317	1,976	No

follicular hyperplasia

Comparison	Diff of Ranks	Q	P<0,05
Col 11 vs Col 35	25,333	3,496	Yes

Col 23 vs Col 35	18,773	2,516	Yes
Col 59 vs Col 35	14,5	2,001	No
Col 47 vs Col 35	10,042	1,386	Do Not Test

hepatitis

Comparison	Diff of Ranks	Q	P<0,05
Col 24 vs Col 36	24,741	3,292	Yes
Col 12 vs Col 36	17,271	2,367	No
Col 60 vs Col 36	16,271	2,23	Do Not Test
Col 48 vs Col 36	12,438	1,704	Do Not Test

Pearson Product Moment Correlation

		Col 65	Col 66	Col 68	Col 69	Col 70
Correlation coefficient	Col 64	-0,886	0,922	0,815	0,877	-0,774
p value(<0,05)		0,0455	0,0258	9,27E-02	0,0509	0,124
Sample number		5	5	5	5	5

		Col 66	Col 68	Col 69	Col 70	Col 71
Correlation coefficient	Col 65	-0,888	-0,925	-0,93	0,975	0,921
p value(<0,05)		0,0445	0,0242	0,0219	0,00484	0,0261
Sample number		5	5	5	5	5

Col 64	lymphoid depletion
Col 65	follicular hyperplasia
Col 66	hepatitis
Col 68	PCR liver

Col 69	PCR cruor
Col 70	ELISA
Col 71	SNT
