# Supplementary material for: Two monoclonal antibodies against glycoprotein Gn protect mice from Rift Valley Fever challenge by cooperative effects
Source: PLoS Negl Trop Dis. 2020 Mar 11;14(3):e0008143. doi: 10.1371/journal.pntd.0008143 (PMC7089562; doi:10.1371/journal.pntd.0008143)

S2 Data file

Gn	Test	Sequence peptide	AA	Gn3	Gn32
>Peptide_01	x	BXEDPHLRNRPGKGHNYIDGMT	1 - 20	0,06	3,75049996
>Peptide_02		BXPGKGHNYIDGMTQEDATCKP	9 - 28	0,0914	3,91709995
>Peptide_03		BXDGMTQEDATCKPVTYAGACS	17 - 36	0,0716	0,1183
>Peptide_04	x	BXTCKPVTYAGACSSFDVLLEK	25 - 44	0,0711	0,0506
>Peptide_05		BXGACSSFDVLLEKGKFPLFQS	33 - 52	0,0825	0,0713
>Peptide_06		BXLLEKGKFPLFQSYAHHRTLL	41 - 60	0,0699	0,064
>Peptide_07	x	BXLFQSYAHHRTLLEAVHDTII	49 - 68	0,0714	0,054
>Peptide_08		BXRTLLEAVHDTIIAKADPPSC	57 - 76	0,0887	0,0996
>Peptide_09		BXDTIIAKADPPSCDLLSAHGN	65 - 84	0,0484	0,0964
>Peptide_10	x	BXPPSCDLLSAHGNPCMKEKLV	73 - 92	0,0584	0,8369
>Peptide_11		BXAHGNPCMKEKLVMKTHCPND	81 - 100	0,0621	0,0744
>Peptide_12		BXEKLVMKTHCPNDYQSAHHLN	89 - 108	0,0539	0,1319
>Peptide_13	x	BXCPNDYQSAHHLNNDGKMASV	97 - 116	0,0633	0,0807
>Peptide_14		BXHHLNNDGKMASVKCPPKYEL	105 - 124	0,0687	0,0679
>Peptide_15		BXMASVKCPPKYELTEDCNFCR	113 - 132	0,0662	0,0983
>Peptide_16	x	BXKYELTEDCNFCRQMTGASLK	121 - 140	0,0509	0,0563
>Peptide_17		BXNFCRQMTGASLKKGSYPLQD	129 - 148	0,0462	0,70310003
>Peptide_18		BXASLKKGSYPLQDLFCQSSED	137 - 156	0,0555	0,0529
>Peptide_19	x	BXPLQDLFCQSSEDDGSKLKTK	145 - 164	0,053	0,36219999
>Peptide_20		BXSSEDDGSKLKTKMKGVCEVG	153 - 172	0,0529	0,0496
>Peptide_21		BXLKTKMKGVCEVGVQALKKCD	161 - 180	0,047	0,059
>Peptide_22	x	BXCEVGVQALKKCDGQLSTAHE	169 - 188	0,0504	0,0574
>Peptide_23		BXKKCDGQLSTAHEVVPFAVFK	177 - 196	0,0581	0,0551
>Peptide_24		BXTAHEVVPFAVFKNSKKVYLD	185 - 204	0,0488	0,0486
>Peptide_25	x	BXAVFKNSKKVYLDKLDLKTEE	193 - 212	0,0449	0,1138
>Peptide_26		BXVYLDKLDLKTEENLLPDSFV	201 - 220	0,0503	0,064
>Peptide_27		BXKTEENLLPDSFVCFEHKGQY	209 - 228	0,0576	0,0815
>Peptide_28	x	BXDSFVCFEHKGQYKGTMDSGQ	217 - 236	0,0503	0,0673
>Peptide_29		BXKGQYKGTMDSGQTKRELKSF	225 - 244	0,0443	0,0603
>Peptide_30		BXDSGQTKRELKSFDISQCPKI	233 - 252	0,0505	0,0734
>Peptide_31	x	BXLKSFDISQCPKIGGHGSKKC	241 - 260	0,0666	0,0807
>Peptide_32		BXCPKIGGHGSKKCTGDAAFCS	249 - 268	0,0535	0,1459
>Peptide_33		BXSKKCTGDAAFCSAYECTAQY	257 - 276	0,0482	0,0937
>Peptide_34	x	BXAFCSAYECTAQYANAYCSHA	265 - 284	0,0519	0,0697
>Peptide_35		BXTAQYANAYCSHANGSGIVQI	273 - 292	0,0559	0,0606
>Peptide_36		BXCSHANGSGIVQIQVSGVWKK	281 - 300	0,0535	0,0706
>Peptide_37	x	BXIVQIQVSGVWKKPLCVGYER	289 - 308	0,0535	0,0755
>Peptide_38		BXVWKKPLCVGYERVVVKRELS	297 - 316	0,055	0,0757
>Peptide_39		BXGYERVVVKRELSAKPIQRVE	305 - 324	0,0534	0,0544
>Peptide_40	x	BXRELSAKPIQRVEPCTTCITK	313 - 332	0,0542	0,0466
>Peptide_41		BXQRVEPCTTCITKCEPHGLVV	321 - 340	0,0473	0,0684
>Peptide_42		BXCITKCEPHGLVVRSTGFKIS	329 - 348	0,051	0,76319999
>Peptide_43	x	BXGLVVRSTGFKISSAVACASG	337 - 356	0,0527	0,0624
>Peptide_44		BXFKISSAVACASGVCVTGSQS	345 - 364	0,0497	0,0805
>Peptide_45		BXCASGVCVTGSQSPSTEITLK	353 - 372	0,0464	0,0979
>Peptide_46	x	BXGSQSPSTEITLKYPGISQSS	361 - 380	0,0492	0,0797
>Peptide_47		BXITLKYPGISQSSGGDIGVHM	369 - 388	0,0477	0,0646
>Peptide_48		BXSQSSGGDIGVHMAHDDQSVS	377 - 396	0,0596	0,0523
>Peptide_49	x	BXGVHMAHDDQSVSSKIVAHCP	385 - 404	0,0462	0,0775
>Peptide_50		BXQSVSSKIVAHCPPQDPCLVH	393 - 412	0,0531	0,083
>Peptide_51		BXAHCPPQDPCLVHDCIVCAHG	401 - 420	0,0521	0,0699
>Peptide_52	x	BXCLVHDCIVCAHGLINYQCHT	409 - 428	0,048	0,0712
>Peptide_53		BXCAHGLINYQCHTALSAFVVV	417 - 436	0,0478	0,1311
>Peptide_54		BXQCHTALSAFVVVFVFSSIAI	425 - 444	0,0452	0,0692
>Peptide_55	x	BXFVVVFVFSSIAIICLAILYR	433 - 452	0,0483	0,0812
>Peptide_56		BXSIAIICLAILYRVLKCLKIA	441 - 460	0,0461	0,052
>Peptide_57		BXILYRVLKCLKIAPRKVLNPL	449 - 468	0,0476	0,0987
>Peptide_58	x	BXLKIAPRKVLNPLMWITAFIR	457 - 476	0,0524	0,0631
>Peptide_59		BXLNPLMWITAFIRWIYKKMVA	465 - 484	0,0484	0,0898
>Peptide_60		BXAFIRWIYKKMVARVADNINQ	473 - 492	0,0471	0,0637
>Peptide_61	x	BXKMVARVADNINQVNREIGWM	481 - 500	0,0454	0,0566
>Peptide_62		BXNINQVNREIGWMEGGQLVLG	489 - 508	0,0545	0,53860003
>Peptide_63		BXIGWMEGGQLVLGNPAPIPRH	497 - 516	0,0515	0,0881
>Peptide_64	x	BXLVLGNPAPIPRHAPIPRYST	505 - 524	0,0548	0,049
>Peptide_65		BXIPRHAPIPRYSTYLMLLLIV	513 - 532	0,0763	0,0808
>Peptide_66		BXPIPRYSTYLMLLLIVSYASA	518 - 537	0,0991	0,1138

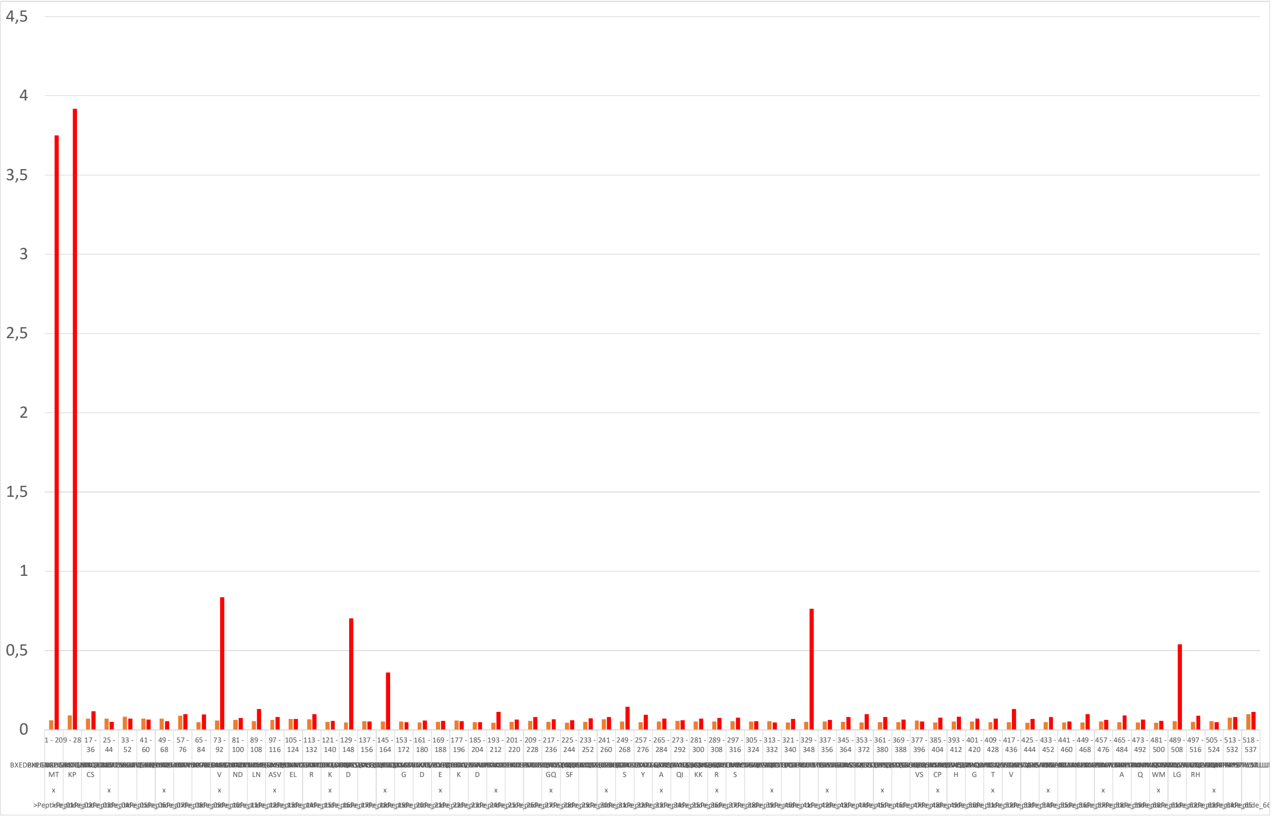

Supplement: S2 Data — (PDF) [file pntd.0008143.s006.pdf]
