# Supplementary material for: Two monoclonal antibodies against glycoprotein Gn protect mice from Rift Valley Fever challenge by cooperative effects
Source: PLoS Negl Trop Dis. 2020 Mar 11;14(3):e0008143. doi: 10.1371/journal.pntd.0008143 (PMC7089562; doi:10.1371/journal.pntd.0008143)
Supplement: S3 Data — (PDF) [file pntd.0008143.s007.pdf]

Fig.1 ELISA fits:

Gn3

	value
**npar**	**4**
**params.bottom**	**4.14837e-02**
**params.top**	**9.13609e-01**
**params.xmid**	**8.61936e-02**
**params.scal**	**1.96978e+00**
params.s	1.00000e+00
GOF	9.93557e-01
weightedGOF	9.99893e-01
stdErr	0.0316608
weighted stdErr	0.02761195
trapezoid	3.06068
Simpson	3.02756
xInfl	0.0861936
yInfl	0.477546
**Log10(EC50)**	**0.1089195**
**EC50**	**1.28505e+00**
**[95%]**	**[1.12456e+00 | 1.47372e+00]**
date (Y-m-d)	2019-04-02
nplr version	0.1.7 (2016-12-25)
R version	3.5.2 (2018-12-20)

Gn32

	value
**npar**	**4**
**params.bottom**	**4.62708e-02**
**params.top**	**9.46424e-01**
**params.xmid**	**-4.27657e-01**
**params.scal**	**1.59518e+00**
params.s	1.00000e+00
GOF	9.89457e-01
weightedGOF	9.99814e-01
stdErr	0.04005807
weighted stdErr	0.03609953
trapezoid	3.64066
Simpson	3.60647
xInfl	-0.427657
yInfl	0.496348
**Log10(EC50)**	**-0.423238**
**EC50**	**3.77365e-01**
**[95%]**	**[3.06354e-01 | 4.65456e-01]**
date (Y-m-d)	2019-04-02
nplr version	0.1.7 (2016-12-25)
R version	3.5.2 (2018-12-20)

Gn3+Gn32

	value
**npar**	**4**
**params.bottom**	**3.79946e-02**
**params.top**	**9.38683e-01**
**params.xmid**	**-3.21513e-01**
**params.scal**	**1.75559e+00**
params.s	1.00000e+00
GOF	9.92697e-01
weightedGOF	9.99881e-01
stdErr	0.0339645
weighted stdErr	0.02937417
trapezoid	3.5009
Simpson	3.46675
xInfl	-0.321513
yInfl	0.488339
**Log10(EC50)**	**-0.3086988**
**EC50**	**4.91248e-01**
**[95%]**	**[4.20256e-01 | 5.76991e-01]**
date (Y-m-d)	2019-04-02
nplr version	0.1.7 (2016-12-25)
R version	3.5.2 (2018-12-20)

Fig. 3 SNT fits:

A MP12:

Gn3

	value
**npar**	**4**
**params.bottom**	**-1.82790e-03**
**params.top**	**1.01021e+00**
**params.xmid**	**1.52787e+00**
**params.scal**	**8.96265e+00**
params.s	1.00000e+00
GOF	9.95093e-01
weightedGOF	9.99906e-01
stdErr	0.03365182
weighted stdErr	0.01681304
trapezoid	0.480254
Simpson	0.474988
xInfl	1.52787
yInfl	0.50419
**Log10(IC50)**	**1.527067**
IC50	3.36563e+01
**[95%]**	**[3.28416e+01 | 3.45e+01]**
date (Y-m-d)	2019-04-04
nplr version	0.1.7 (2016-12-25)
R version	3.5.2 (2018-12-20)

Gn3+Gn32

	value
**npar**	**4**
**params.bottom**	**-9.60328e-04**
**params.top**	**1.01775e+00**
**params.xmid**	**1.39401e+00**
**params.scal**	**5.05766e+00**
params.s	1.00000e+00
GOF	9.407e-01
weightedGOF	9.98574e-01
stdErr	0.1169808
weighted stdErr	0.06540513
trapezoid	0.622314
Simpson	0.61411
xInfl	1.39401
yInfl	0.508393
**Log10(IC50)**	**1.391181**
IC50	2.4614e+01
**[95%]**	**[2.09436e+01 | 2.87556e+01]**
date (Y-m-d)	2019-04-04
nplr version	0.1.7 (2016-12-25)
R version	3.5.2 (2018-12-20)

B

Gn3

	value
**npar**	**4**
**params.bottom**	**-1.53276e-03**
**params.top**	**9.12087e-01**
**params.xmid**	**2.15389e+00**
**params.scal**	**6.09895e+00**
params.s	1.00000e+00
GOF	6.77302e-01
weightedGOF	9.97917e-01
stdErr	0.2728898
weighted stdErr	0.07904551
trapezoid	0.573532
Simpson	0.566938
xInfl	2.15389
yInfl	0.455277
**Log10(IC50)**	**2.167875**
IC50	1.47189e+02
**[95%]**	**[1.05181e+02 | 2.09276e+02]**
date (Y-m-d)	2019-04-02
nplr version	0.1.7 (2016-12-25)
R version	3.5.2 (2018-12-20)

Gn3+Gn32

	value
**npar**	**4**
**params.bottom**	**-3.43922e-04**
**params.top**	**9.99470e-01**
**params.xmid**	**2.00262e+00**
**params.scal**	**5.72752e+00**
params.s	1.00000e+00
GOF	5.64376e-01
weightedGOF	9.95726e-01
stdErr	0.3170623
weighted stdErr	0.1132374
trapezoid	0.604966
Simpson	0.596906
xInfl	2.00262
yInfl	0.499563
**Log10(IC50)**	**2.002748**
IC50	1.00635e+02
**[95%]**	**[6.92891e+01 | 1.45344e+02]**
date (Y-m-d)	2019-04-02
nplr version	0.1.7 (2016-12-25)
R version	3.5.2 (2018-12-20)

EC50 and IC50 values were obtained by fitting the data to a 4-Parameter Logistic Regression model using the nplr package in R.

Frederic Commo and Brian M. Bot (2016). nplr: N-Parameter Logistic Regression. R package version 0.1-7. https://CRAN.R-project.org/package=nplr

R Core Team (2018). R: A language and environment for statistical computing. R Foundation for Statistical Computing, Vienna, Austria. URL https://www.R-project.org/.

H. Wickham. ggplot2: Elegant Graphics for Data Analysis. Springer-Verlag New York, 2016.
