# Supplementary material for: Two monoclonal antibodies against glycoprotein Gn protect mice from Rift Valley Fever challenge by cooperative effects
Source: PLoS Negl Trop Dis. 2020 Mar 11;14(3):e0008143. doi: 10.1371/journal.pntd.0008143 (PMC7089562; doi:10.1371/journal.pntd.0008143)
Supplement: S4 Data — (PDF) [file pntd.0008143.s008.pdf]

S4 Data file

Concentrations used for calculation

SNT MP-12

	Dosis(µg/ml)	F(a)*
Gn3:	25	0.01
	33	0.5
	50	0.99
Gn32:	25	0.01
	33	0.01
	50	0.01
Gn3+Gn32:	15	0.01
	24,6	0.5
	50	0.99

Concentrations used for calculation

SNT 35/74

	Dosis(µg/ml)	F(a)*
Gn3:	133,33	0.01
	147,2	0.5
	177,78	0.99
Gn32:	133,33	0.01
	147,2	0.01
	177,78	0.01
Gn3+Gn32:	57,6	0.01
	100,6	0.5
	144,4	0.99

Concentrations used for calculation

ELISA

	Dosis(µg/ml)	F(a)*
Gn3:	0,5	0.2
	1	0.39
	15	0.99
Gn32:	0,5	0.52
	1	0.88
	15	0.99
Gn3+Gn32:	0,5	0.53
	1	0.79
	15	0.99

* Fraction affected F(a)

CI Data for Drug Combo: combi (Gn3+Gn32 [1:1])

F(a)	CI Value	Total Dose(µg/ml)
0.05	NaN*	17,89
0.1	NaN	19,75
0.15	NaN	21,00
0.2	NaN	21,99
0.25	NaN	22,85
0.3	NaN	23,62
0.35	NaN	24,34
0.4	NaN	25,04
0.45	NaN	25,73
0.5	NaN	26,42
0.55	0,39	27,1354
0.6	0,39	27,8812
0.65	0,40	28,6808
0.7	0,40	29,5608
0.75	0,41	30,5609
0.8	0,41	31,7473
0.85	0,42	33,2456
0.9	0,43	35,3455
0.95	0,45	39,0208
0.97	0,46	41,8664

CI Data for Drug Combo: combi (Gn3+Gn32 [1:1])

F(a)	CI Value	Total Dose(µg/ml)
0.05	NaN	69,88
0.1	NaN	75,39
0.15	NaN	79,02
0.2	NaN	81,86
0.25	NaN	84,29
0.3	NaN	86,4645
0.35	NaN	88,4915
0.4	NaN	90
0.45	NaN	92,3311
0.5	NaN	94,23
0.55	0,31	96,17
0.6	0,32	98,19
0.65	0,32	100,344
0.7	0,33	102,696
0.75	0,34	105,35
0.8	0,34	108,472
0.85	0,35	112,377
0.9	0,36	117,781
0.95	0,38	127,063
0.97	0,40	134,11

CI Data for Drug Combo: combi (Gn3+Gn32 [1:1])

F(a)	CI Value	Total Dose(µg/ml)
0.05	0,81	0,04
0.1	0,81	0,07
0.15	0,81	0,11
0.2	0,81	0,14
0.25	0,81	0,17
0.3	0,81	0,21
0.35	0,81	0,25
0.4	0,81	0,30
0.45	0,81	0,35
0.5	0,82	0,41
0.55	0,82	0,48
0.6	0,82	0,56
0.65	0,83	0,66
0.7	0,83	0,79
0.75	0,84	0,96
0.8	0,84	1,20
0.85	0,85	1,58
0.9	0,87	2,26
0.95	0,90	4,04
0.97	0,93	6,11

DRI Data for Drug Combo: combi (Gn3+Gn32 [1:1])

F(a)	Dose Gn3	Dose Gn32	DRI Gn3	DRI Gn32
0.05	27,59	NaN	3,08379	NaN
0.1	29,21	NaN	2,95747	NaN
0.15	30,26	NaN	2,88	NaN

DRI Data for Drug Combo: combi (Gn3+Gn32 [1:1])

F(a)	Dose Gn3	Dose Gn32	DRI Gn3	DRI Gn32
0.05	137,90	NaN	3,95	NaN
0.1	141	NaN	3,74778	NaN
0.15	143,402	NaN	3,6297	NaN

DRI Data for Drug Combo: combi (Gn3+Gn32 [1:1])

F(a)	Dose Gn3	Dose Gn32	DRI Gn3	DRI Gn32
0.05	0,23	0,03	10,90	1,39
0.1	0,23	0,03	9,26	1,43
0.15	0,23	0,03	8,37	1,46

0.2	31,079	NaN	2,82622	NaN
0.25	31,7698	NaN	2,78108	NaN
0.3	32,3858	NaN	2,74223	NaN
0.35	32,9557	NaN	2,70741	NaN
0.4	33,498	NaN	2,67524	NaN
0.45	34,0263	NaN	2,64475	NaN
0.5	34,5521	NaN	2,61521	NaN
0.55	35,086	Infinit	2,58599	Infinit
0.6	35,6395	Infinit	2,55652	Infinit
0.65	36,2259	Infinit	2,52614	Infinit
0.7	36,8633	Infinit	2,49407	Infinit
0.75	37,5781	Infinit	2,45923	Infinit
0.8	38,4134	Infinit	2,41994	Infinit
0.85	39,4495	Infinit	2,37322	Infinit
0.9	40,8691	Infinit	2,31255	Infinit
0.95	43,2706	Infinit	2,21782	Infinit
0.97	45,0648	Infinit	2,15279	Infinit

0.2	145,026	NaN	3,54326	NaN
0.25	146,381	NaN	3,47342	NaN
0.3	147,575	NaN	3,41353	NaN
0.35	148,668	NaN	3,36004	NaN
0.4	149,697	NaN	3,31075	NaN
0.45	150,692	NaN	3,26416	NaN
0.5	151,672	NaN	3,21914	NaN
0.55	152,659	Infinit	3,17475	Infinit
0.6	153,673	Infinit	3,13008	Infinit
0.65	154,738	Infinit	3,08415	Infinit
0.7	155,884	Infinit	3,03582	Infinit
0.75	157,155	Infinit	2,98348	Infinit
0.8	158,623	Infinit	2,92468	Infinit
0.85	160,419	Infinit	2,85503	Infinit
0.9	162,836	Infinit	2,76507	Infinit
0.95	166,817	Infinit	2,62573	Infinit
0.97	169,708	Infinit	2,53088	Infinit

0.2	0,23	0,03	7,75	1,48
0.25	0,23	0,03	7,28	1,49
0.3	0,23	0,03	6,89	1,51
0.35	0,23	0,03	6,55	1,52
0.4	0,23	0,03	6,26	1,54
0.45	1,05	0,03	5,98	1,55
0.5	1,05	0,03	5,72	1,56
0.55	1,05	0,03	5,48	1,57
0.6	1,05	0,03	5,24	1,58
0.65	1,05	0,03	5,00	1,60
0.7	1,05	0,03	4,76	1,61
0.75	1,05	0,03	4,50	1,63
0.8	1,05	0,03	4,23	1,65
0.85	1,05	1,31	3,92	1,67
0.9	1,05	1,31	3,54	1,70
0.95	1,05	1,31	3,01	1,75
0.97	1,05	1,31	2,68	1,78

*NaN = Not a Number (Value could not be calculated)
