# Supplementary material for: Two monoclonal antibodies against glycoprotein Gn protect mice from Rift Valley Fever challenge by cooperative effects
Source: PLoS Negl Trop Dis. 2020 Mar 11;14(3):e0008143. doi: 10.1371/journal.pntd.0008143 (PMC7089562; doi:10.1371/journal.pntd.0008143)
Supplement: S5 Data — (PDF) [file pntd.0008143.s009.pdf]

		days post infection												
**Treatment**	**Animal designation**	**1**	**2**	**3**	**4**	**5**	**6**	**7**	**8**	**9**	**10**	**11**	**12**	**13**
**PBS group**	25	0	0	**8**										
	26	0	0	0	0	0	**8**							
	27	0	0	**8**										
	28	0	0	0	0	8								
	29	0	0	0	0	0	0	0	0	0	0	0	0	0
	30	0	0	0	0	0	0	0	8					
	31	0	0	0	0	0	**8**							
	32	0	0	0	0	0	**8***							
	33	0	0	0	0	0	0	0	0	0	0	0	0	0
	34	0	0	0	0	0	**8***							
	35	0	0	**8**										
	36	0	0	**8**										
	73	0	0	0	**8**									
	74	0	0	**8***										
	75	0	0	0	0	0	0	0	0	0	0	0	0	0
	76	0	0	0	**8**									
	77	0	0	**8***										
	78	0	0	0	0	0	0	0	0	0	0	0	0	0
	79	0	0	0	**8***									
	80	0	0	0	0	0	0	8						
	81	0	0	**8***										
	82	0	0	0	0	0	0	0	8					
	83	0	0		**8***									
	84	0	0	0	0	0	0	**8***						
	number of surviving animals	24	24	24	17	13	12	8	6	4	4	4	4	4
	total score	0	0	32	16	8	16	16	16	0	0	0	0	0
	mean score	0	0	1,3	0,9	0,6	1,3	2	2,7	0	0	0	0	0
	cumulative score	0	0	2,3	4,1	4,7	7,4	9,4	12,1	12,1	12,1	12,1	12,1	12,1
**Gn3 T1**	97	0	0	0	0	0	0	0	0	0	0	0	0	0
	98	0	0	0	0	0	0	0	0	0	0	0	0	0
	99	0	0	0	0	0	0	0	0	0	0	0	0	0
	100	0	0	0	0	0	0	0	**8**					
	101	0	0	0	0	0	0	0	0	0	0	0	0	0
	102	0	0	0	0	0	0	0	0	0	0	0	0	0
	103	0	0	0	**8***									
	104	0	0	0	0	**8***								
	105	0	0	0	0	0	**8**							
	106	0	0	0	0	0	0	0	0	0	0	0	0	0
	107	0	0	0	0	0	0	0	0	0	0	0	0	0
	108	0	0	**8**										
	number of surviving animals	12	12	12	11	10	9	8	8	7	7	7	7	7
	total score	0	0	8	8	8	8	0	8	0	0	0	0	0
	mean score	0	0	0,7	0,7	0,8	0,9	0	1	0	0	0	0	0
	cumulative score	0	0	0,7	1,4	2,2	3,1	3,1	4,1	4,1	4,1	4,1	4,1	4,1
Gn3 T2	85	0	0	0	0	0	0	0	0	0	0	0	0	0
	86	0	0	0	0	0	0	0	0	0	0	0	0	0
	87	0	0	0	0	0	0	0	0	0	0	0	0	0
	88	0	0	0	0	0	0	0	0	0	0	0	0	0
	89	0	0	0	0	0	0	0	0	0	0	0	0	0
	90	0	0	0	0	0	0	0	0	0	0	0	0	0
	91	0	0	0	0	0	0	0	0	0	0	0	0	0
	92	0	0	0	**8***									
	93	0	0	0	**8**									
	94	0	0	0	**8***									
	95	0	0	0	0	0	8							
	96	0	0	0	**8***									
	number of surviving animals	12	12	12	12	8	8	7	7	7	7	7	7	7
	total score	0	0	0	32	0	8	0	0	0	0	0	0	0
	mean score	0	0	0	2,7	0	1	0	0	0	0	0	0	0
	cumulative score	0	0	0	2,7	2,7	3,7	3,7	3,7	3,7	3,7	3,7	3,7	3,7
Gn3+Gn32 combi T1	1	0	0	0	0	**2**	**8**							
	2	0	0	0	0	0	**2**	0	0	0	0	0	0	0
	3	0	0	0	0	0	0	0	0	0	0	0	0	0
	4	0	0	0	0	0	0	0	0	0	0	0	0	0
	5	0	0	0	0	0	0	0	0	0	0	0	0	0
	6	0	0	0	0	0	**8**							
	7	0	0	0	0	0	0	0	0	0	0	0	0	0
	8	0	0	0	0	0	0	0	0	0	0	0	0	0
	9	0	0	0	0	0	0	0	0	0	0	0	0	0
	10	0	0	0	0	0	0	0	0	0	0	0	0	0
	11	0	0	0	0	0	0	0	0	0	0	0	0	0
	12	0	0	0	0	0	0	0	0	0	0	0	0	0
	number of surviving animals	12	12	12	12	12	12	10	10	10	10	10	10	10
	total score	0	0	0	0	2	18	0	0	0	0	0	0	0
	mean score	0	0	0	0	0,2	1,5	0	0	0	0	0	0	0
	cumulative score	0	0	0	0	0,2	1,7	1,7	1,7	1,7	1,7	1,7	1,7	1,7
	13	0	0	0	0	0	0	0	0	0	0	0	0	0
	14	0	0	0	0	0	0	0	0	0	0	0	0	0
	16	0	0	0	0	0	0	0	0	0	0	0	0	0
	17	0	0	0	0	0	0	0	0	0	0	0	0	0
	18	0	0	0	0	0	**3**	0	0	0	0	0	0	0

Gn3+Gn32 combi T2	19	0	0	0	0	0	0	0	0	0	0	0	0	0
	20	0	0	0	0	0	0	0	0	0	0	0	0	0
	21	0	0	0	0	0	0	0	0	0	0	0	0	0
	22	0	0	0	0	0	0	0	0	0	0	0	0	0
	23	0	0	0	0	0	0	0	0	0	0	0	0	0
	24	0	0	0	0	0	0	0	0	0	0	0	0	0
	number of surviving animals	12	12	12	12	12	12	12	12	12	12	12	12	12
	total score	0	0	0	0	0	3	0	0	0	0	0	0	0
	mean score	0	0	0	0	0,0	0,25	0	0	0	0	0	0	0
	cumulative score	0	0	0	0	0	0,25	0,25	0,25	0,25	0,25	0,25	0,25	0,25
mAb combi group	67	0	0	0	0	0	0	0	0	0	0	0	0	0
	68	0	0	0	0	0	0	0	0	0	0	0	0	0
	69	0	0	0	0	0	0	0	0	0	0	0	0	0
	70	0	0	0	0	0	0	0	0	0	0	0	0	0
	71	0	0	0	0	0	0	0	0	0	0	0	0	0
	72	0	0	0	0	0	0	0	0	0	0	0	0	0
	total score	0	0	0	0	0	0	0	0	0	0	0	0	0
	mean score	0	0	0	0	0	0	0	0	0	0	0	0	0
	cumulative score	0	0	0	0	0	0	0	0	0	0	0	0	0
Gn32 T1 group	121	0	0	0	0	0	0	0	0	0	0	0	0	0
	122	0	0	8*	0									
	123	0	0	8*	0									
	124	0	0	8*	0									
	125	0	0	8*	0									
	126	0	0	8*	0									
	127	0	0	0	0	0	8*							
	128	0	0	0	0	0	0	0	0	0	8			
	129	0	0	0	0	0	8*							
	130	0	0	0	0	0	0	0	0	8				
	131	0	0	0	0	0	0	8						
	132	0	0	0	0	0	0	0	0	8				
	number of surviving animals	12	12	12	7	7	7	5	4	4	2	1	1	1
	total score	0	0	40	0	0	16	8	0	16	8	0	0	0
	mean score	0	0	3,3	0	0	2,3	1,6	0	4	4	0	0	0
	cumulative score	0	0	3,3	3,3	3,3	5,6	7,2	7,2	11,2	15,2	15,2	15,2	15,2
Gn32 T2 group	109	0	0	0	0	0	0	0	0	0	0	0	0	0
	110	0	0	0	0	0	0	0	8					
	111	0	0	8*										
	112	0	0	0	0	0	0	0	0	0	0	0	0	0
	113	0	0	0	0	0	8*							
	114	0	0	0	0	0	0	0	0	0	0	0	0	0
	115	0	0	8*										
	116	0	0	0	0	8								
	117	0	0	8*	0									
	118	0	0	0	8*									
	119	0	0	0	8*									
	120	0	0	0	8*									
	number of surviving animals	12	12	12	9	6	5	4	4	3	3	3	3	3
	total score	0	0	24	24	8	8	8	8	0	0	0	0	0
	mean score	0	0	2	2,7	1,3	1,6	0	2	0	0	0	0	0
	cumulative score	0	0	2	4,7	6	7,6	0	9,6	9,6	9,6	9,6	9,6	9,6

*** found dead**

Cumulative score

Treatment group	days post infection												
	**1**	**2**	**3**	**4**	**5**	**6**	**7**	**8**	**9**	**10**	**11**	**12**	**13**
Gn3 T1	0	0	2,3	4,1	4,7	7,4	9,4	12,1	12,1	12,1	12,1	12,1	12,1
Gn3 T2	0	0	0,7	1,4	2,2	3,1	3,1	4,1	4,1	4,1	4,1	4,1	4,1
Gn3+Gn32 combi T1	0	0	0	2,7	2,7	3,7	3,7	3,7	3,7	3,7	3,7	3,7	3,7
Gn3+Gn32 combi T2	0	0	0	0	0,2	1,7	1,7	1,7	1,7	1,7	1,7	1,7	1,7
mAb combi group	0	0	0	0	0	0,25	0,25	0,25	0,25	0,25	0,25	0,25	0,25
Gn32 T1 group	0	0	3,3	3,3	3,3	5,6	7,2	7,2	11,2	15,2	15,2	15,2	15,2
Gn32 T2 group	0	0	2	4,7	6	7,6	7,6	9,6	9,6	9,6	9,6	9,6	9,6
