# Supplementary material for: Two monoclonal antibodies against glycoprotein Gn protect mice from Rift Valley Fever challenge by cooperative effects
Source: PLoS Negl Trop Dis. 2020 Mar 11;14(3):e0008143. doi: 10.1371/journal.pntd.0008143 (PMC7089562; doi:10.1371/journal.pntd.0008143)
Supplement: S6 Data — (PDF) [file pntd.0008143.s010.pdf]

Treatment	Animal number	necropsy (dpi)	PCR values [copies/mg]			Virustitration [TCID50/ml]			ELISA [S/N%]	Serum neutralisation test [titer]	IHC (NP antigen)				Histopathology			
			cruor	liver	brain	cruor	liver	brain			loung	spleen	liver	brain	lymphoid depletion	follicular hyperplasia	hepatitis	encephalitis
PBS group	25	3	225000	164507,0423	25	56234,13252	3.162.278	3.162	100,1851806	<10	1	2	3	0	2	0	3	0
	26	6	8930	179693,2515	10,90689655	5623,413252	1000000	316	80,09629638	<10	2	2	3	0	2	0	3	0
	27	3	181000	273770,9497	2609,665428	31622,7766	562.341	1.778	n.t.	n.t.	0	1	3	0	1	0	3	0
	28	5	84100	277087,3786	567,9012346	5623,413252	562.341	316	105,3185179	<10	0	n.t.	3	0	n.t.	n.t.	3	0
	29	13	296	53,24074074	33,09859155	0	0	0	93,42962559	<10	0	0	0	0	0	0	0	0
	30	8	53,8	532,1243523	285000	0	0	3.162.278	23,57036937	10	0	1	0	2	2	0	2	1
	31	6	219	39563,90977	182,3076923	10000	5.623	3.162	23,62962964	n.t.	0	0	3	0	2	0	3	0
	32	6	n.t.	127480,6202	7,54185022	n.t.	100.000	3.162	n.t.	n.t.	0	0	3	0	1	0	3	0
	33	13	0	9,524861878	0,929378531	0	0	0	101,3481422	<10	0	0	0	0	0	0	1	0
	34	6	2120	188626,3736	244,6666667	178	0	6.761			0	0	3	0	2	0	3	0
	35	3	54800	931658,2915	977,7777778	562341,3252	1.778.279	1.778	100,4296303	<10	0	1	3	0	1	0	3	0
	36	3	37700	21286,48649	228,5608856	6.761	316227,766	5.623	n.t.	n.t.	0	2	3	0	1	0	3	0
	73	4	115000	398192,7711	1721,081081	100000	1000000	316	103,4074076	<10	2	2	3	0	2	0	3	0
	74	3	17600	697538,4615	199,5512821	147910,8388	1000000	676	102,7925879	<10	0	0	3	0	1	0	3	0
	75	13	69,6	125,2606635	0	0	0	0	95,91111261	<10	0	0	0	0	0	0	0	0
	76	4	481000	171932,2034	1847,619048	56234,13252	1.778.279	676	104,051853	<10	0	1	3	0	2	0	3	0
	77	3	246000	240959,596	1189,189189	316227,766	10.000	562	103,3777731	n.t.	0	1	3	0	1	0	3	0
	78	13	98,5	602	0	0	1.000	0	103,5777798	<10	0	0	0	0	0	0	0	0
	79	4	n.t.	794491,5254	242,6989619	n.t.	1.778	100	n.t.	n.t.	1	2	3	0	2	0	3	0
	80	7	854	2336,681223	12,72463768	178	0	1.778	20,31111019	10	0	0	2	0	1	0	3	1
	81	3	139000	907602,3392	51,72794118	562	31622,7766	316	111,5407394	n.t.	0	0	3	0	2	0	3	0
	82	8	60,5	14,45488722	96,2	0	0	562	36,45925767	30	0	0	0	0	0	0	0	0
	83	4	8710	273369,5652	45,4516129	31622,7766	63.096	1.000	87,70370329	<10	0	2	3	0	2	0	3	0
	84	7	n.t.	4058,525346	286,6096866	n.t.	0	3.162	n.t.	n.t.	0	0	3	0	2	0	3	0
Gn3 T1	97	13	0	11,67167382	7,014662757	0	0	0	22,24444421	<10	0	0	0	0	0	1	0	0
	98	13	0	26,03399433	2,175908222	0	0	0	30,37036957	30	0	0	0	0	0	2	1	0
	99	13	0	3,597972973	0	0	0	0	12,29629561	20	0	0	0	0	0	1	1	0
	100	8	0	1,836936937	0	0	0	0	47,73333253	15	0	0	0	0	0	1	1	0
	101	13	0	0,843786982	12,51351351	0	0	0	24,22222121	20	0	0	0	1	0	1	0	2
	102	13	0	1,343161094	0	0	0	0	18,68888813	30	0	0	0	0	0	0	0	0
	103	4	17395,12855	46265,06024	8,993576017	10000	630.957	3.162	101,7333295	<10	1	3	3	0	3	0	3	0
	104	5	34349,65035	407333,3333	18,49329759	3162,27766	630.957	1.778	100,8444433	<10	1	2	3	0	2	0	3	0
	105	6	760,936133	87296,2963	28,38125	0	31622,7766	562	30,96296335	10	0	0	3	0	2	0	3	0
	106	13	0	4,207207207	21,48241206	0	0,0	56,2	23,77777808	15	0	0	0	0	0	0	0	0
	107	13	0	8,124579125	0	0	0,0	0	104,7333328	<10	0	0	0	0	0	0	0	0
	108	3	4,381713393	302760	360,9284333	3162,27766	630.957	1.778	114,2148157	<10	0	1	3	0	1	0	3	0
Gn3 T2	85	13	0	1,041245136	48,68035191	0	0	562	45,13333382	30	0	0	0	0	0	0	0	0
	86	13	0	0	291,6883117	0	0	56	24,83703649	15	0	0	0	0	0	0	0	1
	87	13	0	0	11,15966387	0	0	0	17,94074058	15	0	0	0	1	0	2	0	2
	88	13	0	0	0	0	0	0	48,39999943	10	0	0	0	0	0	0	1	0
	89	13	0	0	12,70322581	0	0	177,8	30,09629638	15	0	0	0	0	0	2	1	0
	90	13	0	0	0	0	0	0	45,04444343	30	0	0	0	0	0	1	1	0
	91	13	0	2,748657718	0	0	0	0	33,14814686	20	0	0	0	0	0	0	0	0
	92	4	161841,0853	37251,08225	389,1647856	31622,7766	100.000	1.778	n.t.	n.t.	1	2	3	0	0	0	3	0
	93	4	39177,16827	60487,80488	151,1241218	3162,27766	316227,766	562	108,3407383	<10	0	2	3	0	1	0	3	0
	94	4	n.t.	296341,4634	64,38735178	n.t.	316227,766	177,8	n.t.	n.t.	1	2	3	0	1	0	3	0
	95	6	3053,100775	1063698,63	96,04166667	31622,7766	1.995.262	316	87,83703225	<10	1	3	3	0	2	0	3	0
	96	4	n.t.	573275,8621	702,1126761	n.t.	63.096	1.000	n.t.	n.t.	1	2	3	0	0	0	3	0
Gn3+Gn32 combi T1	1	6	6709,894332	59025,06964	44,89345064	562.341	316227,77	10000	61,76296319	<10	0	0	3	0	0	0	3	0
	2	13	18,4	25,75769231	2,101746725	0	0	0	23,25185099	10	0	0	0	0	0	1	1	0
	3	13	0	3,145928339	150,0458716	0	0	100	40,61481616	60	0	0	0	0	0	1	1	2
	4	13	0	0	0	0	0	0	33,93333207	20	0	0	0	0	0	1	1	0
	5	13	0	0	8,355311355	0	0	0	98,26666164	10	0	0	0	0	0	0	0	0
	6	6	1390	61000	20,984375	316,227766	31.623	0	33,36296199	15	0	2	3	1	2	0	3	0
	7	13	0	4,634920635	6,989304813	0	0	0	36,45925767	40	0	0	0	0	0	1	0	1
	8	13	0	0	0	0	0	0	23,69629633	20	0	0	0	0	0	1	1	0
	9	13	0	0	0,583076923	0	0	0	33,70370408	45	0	0	0	0	0	1	1	0
	10	13	0	0	0	0	0	0	30,53333308	30	0	0	0	0	0	2	1	0
	11	13	0	0	80,53571429	0	0	0	30,33333301	20	0	0	0	0	0	1	1	0
	12	13	0	0	0	0	0	0	38,0740733	20	0	0	0	0	0	1	0	0
Gn3+Gn32 combi T2	13	13	8,630841121	0	0	0	0	0	44,75555517	20	0	0	0	0	0	1	0	0
	14	13	0	0	0	0	0	0	99,40740622	<10	0	0	0	0	0	0	0	0
	16	13	0	0	1,338235294	0	0	0	21,92592582	15	0	0	1	0	0	1	2	0
	17	13	0	0	0	0	0	0	94,28888614	<10	0	0	0	0	0	0	0	0
	18	13	0	0	4,5	0	0	0	22,14814739	40	0	0	0	1	0	0	1	1
	19	13	0	0	0	0	0	0	89,53332743	15	0	0	0	0	0	0	0	0
	20	13	0	0	0	0	0	0	27,11851902	40	0	0	0	0	0	1	1	0
	21	13	0	0	13,93087558	0	0	0	26,42222199	30	0	0	0	0	0	2	1	0
	22	13	0	0	0	0	0	0	104,6370382	<10	0	0	0	0	0	0	1	0
	23	13	0	0	0	0	0	0	24,58518479	30	0	0	0	0	0	1	1	0

negative
positive
borderline

n.t. not tested

	24	13	0	0	0	0	0	0	22,79999922	15	0	0	0	0	0	1	0	0
Gn32 T1	121	13	0	0,003	0,034339064	n.t.	n.t.	n.t.	53,49164606	n.t.	n.t.	n.t.	n.t.	n.t.	n.t.	n.t.	n.t.	n.t.
	122	3	16205,90757	2771338,763	5,489620142	n.t.	n.t.	n.t.	97,8310268	n.t.	n.t.	n.t.	n.t.	n.t.	n.t.	n.t.	n.t.	n.t.
	123	3	7216,876096	106269,6053	29,14575592	n.t.	n.t.	n.t.	95,39138583	n.t.	n.t.	n.t.	n.t.	n.t.	n.t.	n.t.	n.t.	n.t.
	124	3	n.t.	2578508,46	6,598449783	n.t.	n.t.	n.t.	n.t.	n.t.	n.t.	n.t.	n.t.	n.t.	n.t.	n.t.	n.t.	n.t.
	125	3	n.t.	1356641,744	0,004	n.t.	n.t.	n.t.	n.t.	n.t.	n.t.	n.t.	n.t.	n.t.	n.t.	n.t.	n.t.	n.t.
	126	3	55458,51054	1002566,005	5,474343058	n.t.	n.t.	n.t.	105,2943053	n.t.	n.t.	n.t.	n.t.	n.t.	n.t.	n.t.	n.t.	n.t.
	127	6	n.t.	370607,9372	0,1933242	n.t.	n.t.	n.t.	n.t.	n.t.	n.t.	n.t.	n.t.	n.t.	n.t.	n.t.	n.t.	n.t.
	128	10	0	0,052916468	8,368945543	n.t.	n.t.	n.t.	30,66873533	n.t.	n.t.	n.t.	n.t.	n.t.	n.t.	n.t.	n.t.	n.t.
	129	6	131,7149124	670953,7716	0	n.t.	n.t.	n.t.	47,77508977	n.t.	n.t.	n.t.	n.t.	n.t.	n.t.	n.t.	n.t.	n.t.
	130	9	0	0,005	3,095380466	n.t.	n.t.	n.t.	39,56837194	n.t.	n.t.	n.t.	n.t.	n.t.	n.t.	n.t.	n.t.	n.t.
	131	7	0	0,089581815	8129,388126	n.t.	n.t.	n.t.	30,18513729	n.t.	n.t.	n.t.	n.t.	n.t.	n.t.	n.t.	n.t.	n.t.
	132	9	0	0	0,103771678	n.t.	n.t.	n.t.	35,03554725	n.t.	n.t.	n.t.	n.t.	n.t.	n.t.	n.t.	n.t.	n.t.
Gn32 T2	109	13	0	0,038646599	1,959444002	n.t.	n.t.	n.t.	24,73564569	n.t.	n.t.	n.t.	n.t.	n.t.	n.t.	n.t.	n.t.	n.t.
	110	8	0	0	6,455519712	n.t.	n.t.	n.t.	22,23104386	n.t.	n.t.	n.t.	n.t.	n.t.	n.t.	n.t.	n.t.	n.t.
	111	3	996,5760339	88914,92316	1,263923116	n.t.	n.t.	n.t.	106,9616349	n.t.	n.t.	n.t.	n.t.	n.t.	n.t.	n.t.	n.t.	n.t.
	112	13	0	0	0	n.t.	n.t.	n.t.	17,19296929	n.t.	n.t.	n.t.	n.t.	n.t.	n.t.	n.t.	n.t.	n.t.
	113	6	6,46647696	408,3090841	0,070249883	n.t.	n.t.	n.t.	n.t.	n.t.	n.t.	n.t.	n.t.	n.t.	n.t.	n.t.	n.t.	n.t.
	114	13	0	0	0	n.t.	n.t.	n.t.	20,3399618	n.t.	n.t.	n.t.	n.t.	n.t.	n.t.	n.t.	n.t.	n.t.
	115	3	21948,40109	5644859,958	82,17787177	n.t.	n.t.	n.t.	107,6617728	n.t.	n.t.	n.t.	n.t.	n.t.	n.t.	n.t.	n.t.	n.t.
	116	5	78,6229674	9478,36	0,002	n.t.	n.t.	n.t.	87,79818993	n.t.	n.t.	n.t.	n.t.	n.t.	n.t.	n.t.	n.t.	n.t.
	117	3	n.t.	5133810,682	0,088776344	n.t.	n.t.	n.t.	n.t.	n.t.	n.t.	n.t.	n.t.	n.t.	n.t.	n.t.	n.t.	n.t.
	118	4	3312,280101	1315727,465	0,246727063	n.t.	n.t.	n.t.	103,2805206	n.t.	n.t.	n.t.	n.t.	n.t.	n.t.	n.t.	n.t.	n.t.
	119	4	31622,41747	6034712,999	14,44	n.t.	n.t.	n.t.	112,5482652	n.t.	n.t.	n.t.	n.t.	n.t.	n.t.	n.t.	n.t.	n.t.
	120	4	2333,157671	415333,7627	1,358376969	n.t.	n.t.	n.t.	104,8251432	n.t.	n.t.	n.t.	n.t.	n.t.	n.t.	n.t.	n.t.	n.t.

n.t. not tested
