# Supplementary material for: Two monoclonal antibodies against glycoprotein Gn protect mice from Rift Valley Fever challenge by cooperative effects
Source: PLoS Negl Trop Dis. 2020 Mar 11;14(3):e0008143. doi: 10.1371/journal.pntd.0008143 (PMC7089562; doi:10.1371/journal.pntd.0008143)
Supplement: S7 Data — (PDF) [file pntd.0008143.s011.pdf]

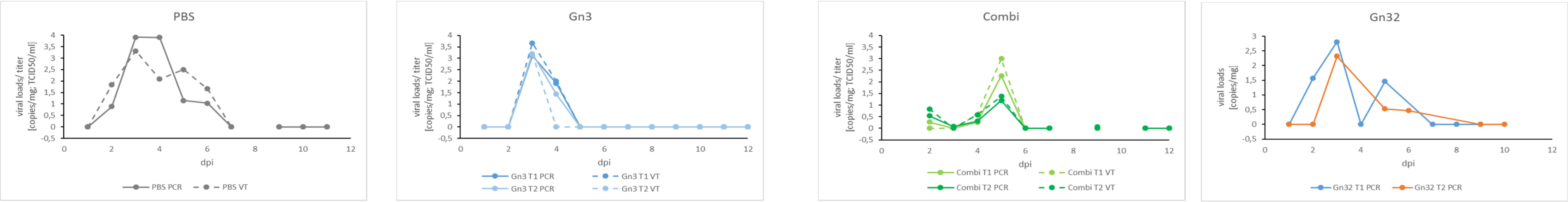


PCR log[copies/mg]
(virus titration log[TCID50/ml])

negative (green)
positive (red)
not tested (grey)

| | animal number | necropsied dpi | 1dpi | 2dpi | 3dpi | 4dpi | 5dpi | 6dpi | 7dpi | 8dpi | 9dpi | 10dpi | 11dpi | 12dpi |
|---|---|---|---|---|---|---|---|---|---|---|---|---|---|---|
| Gn3+Gn32combi T1 | 1 | 6 | | | | | 3,95(5,25) | | | | | | | |
| | 2 | 13 | | | | | 2,84(3,75) | | | | 0 | | | |
| | 3 | 13 | | | | | 0 | | | | 0 | | | |
| | 4 | 13 | | 0,82(0) | | | | | | | | | | |
| | 5 | 13 | | 0 | | | | 0 | | | | | | |
| | 6 | 6 | | 0 | | | | | | | | | | |
| | 7 | 13 | | | 0 | | | | 0 | | | | 0 | |
| | 8 | 13 | | | 0 | | | | 0 | | | | 0 | |
| | 9 | 13 | | | 0 | | | | 0 | | | | 0 | |
| | 10 | 13 | | | | 0 | | | | | | | | 0 |
| | 11 | 13 | | | | 0,39(1,75) | | | | | | | | 0 |
| | 12 | 13 | | | | 0,4(0) | | | | | | | | 0 |
| Gn3+Gn32 combi T2 | 13 | 13 | | | | | 2,38(2,75) | | | | 0,14(0) | | | |
| | 14 | 13 | | | | | 0 | | | | 0 | | | |
| | 16 | 13 | | 0 | | | | | | | | | | |
| | 17 | 13 | | 0 | | | | 0 | | | | | | |
| | 18 | 13 | | 1,62(2,5) | | | | | | | | | | |
| | 19 | 13 | | | 0 | | | | 0 | | | | 0 | |
| | 20 | 13 | | | 0,25(0) | | | | 0 | | | | 0 | |
| | 21 | 13 | | | 0 | | | | 0 | | | | 0 | |
| | 22 | 13 | | | | 0 | | | | | | | | 0 |
| | 23 | 13 | | | | 0,93(1,75) | | | | | | | | 0 |
| | 24 | 13 | | | | 0 | | | | | | | | 0 |
| | animal number | necropsied dpi | 1dpi | 2dpi | 3dpi | 4dpi | 5dpi | 6dpi | 7dpi | 8dpi | 9dpi | 10dpi | 11dpi | 12dpi |
| PBS | 25 | 3 | 0 | | | | | | | | | | | |
| | 26 | 6 | 0 | | | | 2,3(5) | | | | | | | |
| | 27 | 3 | 0 | | | | | | | | | | | |
| | 28 | 5 | | 0 | | | | | | | | | | |
| | 29 | 13 | | 0 | | | | 1,75(3,25) | | | | 0 | | |
| | 30 | 8 | | 3,24(5) | | | | 1,34(1,75) | | | | | | |
| | 31 | 6 | | | | | | | | | | | | |
| | 32 | 6 | | | | | | | | | | | | |
| | 33 | 13 | | | 1,54(0) | | | | 0 | | | | 0 | |
| | 34 | 6 | | | | | | | | | | | | |
| | 35 | 3 | | | | | | | | | | | | |
| | 36 | 3 | | | | | | | | | | | | |
| | 73 | 4 | 0 | | | | | | | | | | | |
| | 74 | 3 | 0 | | | | | | | | | | | |
| | 75 | 13 | 0 | | | | 0 | | | | 0 | | | |
| | 76 | 4 | | 0,93(1,75) | | | | | | | | | | |
| | 77 | 3 | | 1,14(4,25) | | | | | | | | | | |
| | 78 | 13 | | 0 | | | | 0 | | | | 0 | | |
| | 79 | 4 | | | 3,96(5,25) | | | | | | | | | |
| | 80 | 7 | | | 5(4,25) | | | | | | | | | |
| | 81 | 3 | | | 5,15(3,75) | | | | | | | | | |
| | 82 | 8 | | | | 2,66(0) | | | | | | | | |
| | 83 | 4 | | | | 5,14(3) | | | | | | | | |
| | 84 | 7 | | | | 3,92(3,25) | | | | | | | | |
| | animal number | necropsied dpi | 1dpi | 2dpi | 3dpi | 4dpi | 5dpi | 6dpi | 7dpi | 8dpi | 9dpi | 10dpi | 11dpi | 12dpi |
| | 85 | 13 | 0 | | | | 0 | | | | 0 | | | |
| | 86 | 13 | 0 | | | | 0 | | | | 0 | | | |
| | 87 | 13 | 0 | | | | 0 | | | | | | | |

| | | | | | | | | | | | | | | |
|---|---|---|---|---|---|---|---|---|---|---|---|---|---|---|
| | 88 | 13 | | 0 | | | | 0 | | | | 0 | | |
| | 89 | 13 | | 0 | | | | 0 | | | | 0 | | |
| Gn3 T2 | 90 | 13 | | 0 | | | | | | | | 0 | | |
| | 91 | 13 | | | 0 | | | | 0 | | | | | |
| | 92 | 4 | | | 5,77(5,5) | | | | | | | | | |
| | 93 | 4 | | | 3,84(4) | | | | | | | | | |
| | 94 | 4 | | | | | | | | | | | | |
| | 95 | 6 | | | | 1,44(0) | | | | | | | | |
| | 96 | 4 | | | | | | | | | | | | |
| | 97 | 13 | 0 | | | | 0 | | | | 0 | | | |
| | 98 | 13 | 0 | | | | 0 | | | | 0 | | | |
| | 99 | 13 | 0 | | | | 0 | | | | 0 | | | |
| | 100 | 8 | | 0 | | | | 0 | | | | | | |
| | 101 | 13 | | 0 | | | | 0 | | | | 0 | | |
| Gn3 T1 | 102 | 13 | | 0 | | | | 0 | | | | | | |
| | 103 | 4 | | | 3,49(4) | | | | | | | | | |
| | 104 | 5 | | | 2,33(4) | | | | | | | | | |
| | 105 | 6 | | | 3,51(3) | | | | | | | | | |
| | 106 | 13 | | | | 1,91(2) | | | | | | | | 0 |
| | 107 | 13 | | | | | | | | 0 | | | | 0 |
| | 108 | 3 | | | | | | | | | | | | |
| | animal number | necropsied dpi | 1dpi | 2dpi | 3dpi | 4dpi | 5dpi | 6dpi | 7dpi | 8dpi | 9dpi | 10dpi | 11dpi | 12dpi |
| | 109 | 13 | 0 | | | | 0,78 | | | | 0 | | | |
| | 110 | 8 | 0 | | | | 0,27 | | | | | | | |
| | 111 | 3 | 0 | | | | | | | | | | | |
| | 112 | 13 | | 0 | | | | 0 | | | | 0 | | |
| | 113 | 6 | | 0,01 | | | | 1,41 | | | | | | |
| Gn32 T2 | 114 | 13 | | 0 | | | | 0 | | | | 0 | | |
| | 115 | 3 | | | | | | | | | | | | |
| | 116 | 5 | | | 2,32 | | | | | | | | | |
| | 117 | 3 | | | | | | | | | | | | |
| | 118 | 4 | | | | | | | | | | | | |
| | 119 | 4 | | | | | | | | | | | | |
| | 120 | 4 | | | | | | | | | | | | |
| | 121 | 13 | 0 | | | | 1,46 | | | | 0 | | | |
| | 122 | 3 | 0 | | | | | | | | | | | |
| | 123 | 3 | | | | | | | | | | | | |
| | 124 | 3 | | 1,19 | | | | | | | | | | |
| | 125 | 3 | | 2,52 | | | | | | | | | | |
| Gn32 T1 | 126 | 3 | | 1 | | | | | | | | | | |
| | 127 | 6 | | | 4,34 | | | | | | | | | |
| | 128 | 10 | | | -0,06 | | | | 0 | | | | | |
| | 129 | 6 | | | 4,14 | | | | | | | | | |
| | 130 | 9 | | | | -0,62 | | | | 0 | | | | |
| | 131 | 7 | | | | -0,43 | | | | | | | | |
| | 132 | 9 | | | | 0 | | | | 0 | | | | |
